# The protein corona at the nano-bio interface: the need for standardized methodology and opportunities for neurodegenerative disease intervention

**DOI:** 10.1039/d6ra01255h

**Published:** 2026-05-21

**Authors:** Getasew Shitaye, Martina Dragone, Zewdie Mekonnen, Awet Ghebretinsae Tewelde, Maria Della Valle, Gaetano Caputo, Mohammadhossein Mosalaeizadehyazd, Gianluca D'Abrosca, Luigi Russo, Roberto Fattorusso, Carla Isernia, Gaetano Malgieri

**Affiliations:** a Department of Environmental, Biological and Pharmaceutical Science and Technology, University of Campania “Luigi Vanvitelli” Caserta Italy getasewshitaye.ayalew@unicampania.it; b Department of Biochemistry, College of Medicine and Health Sciences, Bahir Dar University Bahir Dar Ethiopia getasew.shitaye@bdu.edu.et; c Department of Human Sciences, Link Campus University Roma Italy; d Institute of Biostructures and Bioimaging, CNR Naples Italy

## Abstract

The “biological identity” of a nanoparticle is determined not only by its surface but by the complex protein corona (PC) it forms when entering a biological system. There have been significant advances in nanoparticle (NP) design that overcome various barriers, however, the protein corona remains a major challenge in the field of nanotechnology. Despite the use of various analytical techniques and a wide range of experimental conditions in protein corona research, there is a critical need for standardized, high-resolution methods to achieve the most accurate and comprehensive characterization of the protein corona. Improving technical transparency and reproducibility will help develop a more predictable framework for nanomedicine. This review first highlights how the physicochemical properties of NPs and the biological environment influence PC formation. Next, we emphasize the urgent need for standardized methodologies and techniques for their characterization to enhance understanding of protein corona formation and to increase reproducibility in nanomedicine studies. Additionally, to explore the untapped potential of NPs in either accelerating or inhibiting the pathological hallmarks of neurodegenerative diseases, this review summarizes how NPs modulate protein aggregation and fibril formation, and discusses the impact of PC on the process of amyloid fibrillation.

## Abstract 2

This review analyzes the protein corona at the nano-bio interface and proposes a standardized methodological framework. We explore how nanoparticles can accelerate or inhibit neurodegenerative pathology, turning the PC into a functional tool.

## Graphical abstract

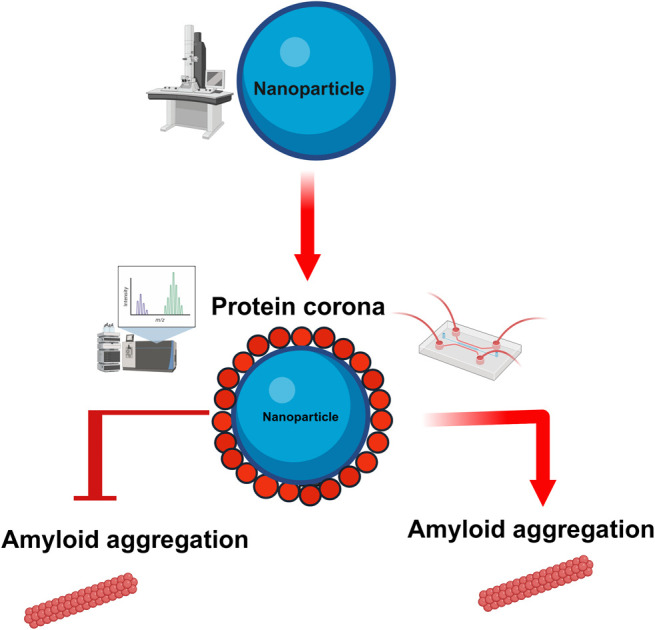

## 1 Introduction

Any material that falls within the size range of one to several tens of nanometers, regardless of whether it is organic, inorganic, or a combination of both, can be classified as a nanoparticle (NP).^1,2^ Interestingly, NPs are comparable in size to biological macromolecules, and tend to be quite stable with low-to-null systemic toxicity, which makes them a promising area of research for a variety of applications.^3,4^ These nanosystems can be finely tuned in their peculiar physico-chemical properties, such as shape, size, charge, hydrophobicity, and surface features which can all be precisely controlled to achieve desired outcomes.^5^ Furthermore, nanoparticle-based therapies have the potential to offer several benefits, including more precise drug delivery, improved solubility, prevention of drug degradation, enhanced therapeutic efficacy, and reduced immune response.^6–8^

It is important to consider the complexity of the environment in which nanoparticles function, as this can greatly influence their behavior and performance. One key difference between well-controlled *in vitro* experiments and *in vivo* applications is the presence of a complex mixture of extracellular proteins.^9^ Despite numerous preclinical researches on nanomedicine, their clinical translation still limited. In fact, in protein-rich biological fluids, NPs quickly acquire a layer of proteins on the surface, forming the so-called “protein corona” (PC).^10^ Protein corona gives to NPs a new biological identity that determines various biological responses including cellular uptake, bio-distribution, pharmacokinetics, cell interaction, and toxicity.^11,12^ For instance, growing evidences indicate that protein corona mediates the impact of nanomaterials in the fibrillation process.^13–15^ Therefore, protein corona formation remains the major bottleneck in the application of nanoparticles in specific drug delivery.

To date, numerous analytical techniques and a diverse set of experimental conditions are being used to properly characterize the parameters that pertain to the nanoparticle, to the bio-system, and to the interaction surfaces of the nanoparticle-protein corona. However, there are still challenges in this research area, that mainly regard the methods providing the most precise and comprehensive characterization of the protein corona.

Here in this review, first, we highlight the influence of physicochemical properties of NPs and biological environment on PC formation. Then, we outline the pressing need of more standardized methodology and techniques for their characterization to further drive our understanding of protein corona formation and improve reproducibility in nanomedicine reports. Moreover, despite the fact that the NPs interaction with neurodegenerative diseases causing proteins have been a subject of extensive research in the field of nanomedicine, the influence of protein corona–NP interaction in the fibrillation process seems at its early stage. NPs penetrate biological barriers and accumulate within the brain, triggering oxidative stress, disrupting the blood–brain barrier (BBB), promoting chronic neuroinflammation, and accelerating the aggregation of hallmark proteins such as amyloid-β, tau, and α-synuclein. In contrast, engineered platforms such as nanobodies, nanozymes, and advanced NPs offer transformative therapeutic and diagnostic capabilities, highlighting the precision of modern nanomedicine. Therefore, building on these crucial findings this review also summarizes the role of NPs in modulating protein aggregation and fibril formation. Furthermore, monitoring the fibrillation process of amyloidal proteins in the presence of corona-coated NPs, rather than NPs only, could help to achieve a more reliable and predictable outcome. In this context the review introduces the impact of PC in the process of amyloid fibrillation and details the role of NP–PC complex as either an anti-fibrillatory or a pro-fibrillatory role in the fibrillation process of amyloid proteins. Finally, the review concludes with several mechanistic models describing how the protein corona competes with amyloidogenic proteins for the NP surface. These includes kinetic trapping, biomolecular shielding, steric hindrance (or exclusion) and monomer sequestration.

## 2 Protein corona formation, composition and its dynamic exchange

Protein corona is the biomolecular shell formed on the surface of NPs. Based on the binding force of proteins to nanomaterials, protein corona is categorized into “hard corona” and “soft corona”. The inner layer of protein corona is composed of tightly bound proteins termed “hard corona” while the outer layer is composed of weakly bound and rapidly exchanging layer of proteins termed “soft corona”.^10^

When NPs are coated with proteins, they acquire new properties such as increased particle dimension, altered zeta potential and aggregation size, and new functional groups, among others.^16^ The protein corona composition and interaction with NPs will influence their biological fate in either negative or positive way impacting various stages such as blood circulation, accumulation and penetration at targeting sites, cellular uptake, and interactions of NPs with receptors on immune cells.^17,18^ Furthermore, the formation of protein corona may cause changes in the secondary structure of proteins so that they lose their actual biological activity.^19^

The evolving PC-nanoparticle interaction is a highly complex phenomenon that relies on the dynamicity of the biological environment and on NPs physicochemical properties. *In vitro* and *in vivo* studies demonstrated that protein corona formation is affected by the nanoparticle size, surface charge,^20^ surface chirality^21^ and curvature,^22^ and by biological environment, and nature of fluid dynamics,^23^ as well as by temperature,^24^ time of exposure^25^ and techniques of preparation. Furthermore, injected NPs traverse vascular networks, and the vascular features (branching and diameter) by themselves can impact the protein corona composition.^26^

### 2.1. Influence of nanoparticle properties on protein corona formation

**Fig. 1:**
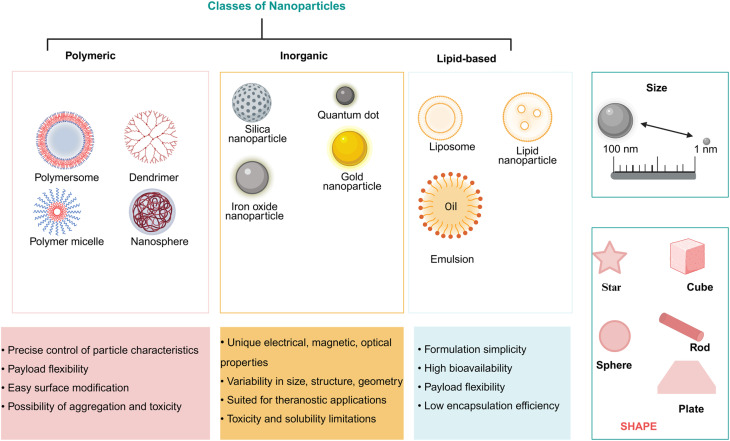
Cartoon representing the different classes of Nanoparticles (NPs). Each class has numerous broad advantages and disadvantages regarding cargo delivery. The scheme provides a representative overview of the three primary classes in current nanomedicine: polymeric NPs (*e.g.*, polymeric micelles, polymersomes, nanospheres), engineered for controlled drug release; inorganic NPs (*e.g.*, gold, silica, iron oxide), utilized as high-performance contrast agents due to their unique physical properties; and lipid-based NPs (*e.g.*, liposomes, lipid nanoparticles), a prominent class of nanocarriers for nucleic acid (mRNA) vaccines and the encapsulation of hydrophobic therapeutics. The size scale (right) indicates the importance of the nanometric range in overcoming biological barriers and dictating circulation time. The shapes (bottom) illustrate diverse morphologies engineered to move from spherical geometries to non-spherical shapes (*e.g.*, rods, stars, or plates) to improve cellular interactions and targeting efficiency.

The optimization of NPs features allows the customization of their functionalities to suit various biomedical applications. Thus, to fully utilize and appropriately modulate the distinctive characteristics of nanoscale materials during their delivery to the target tissues, it is very important to achieve a better understanding of how NP physicochemical properties such as the size, shape, and/or surface chemistry, affect the formation and composition of protein corona (Fig. 1).^27^

#### 2.1.1. Type of nanoparticle (composition)

NPs can be categorized into several groups based on their origin, size and chemical characteristics.^28^ Among hundreds of NP types, metal oxide NPs and metal-based NPs are commonly used in nearly 30% of protein corona research reports. Others, such as polymer based, lipid based, carbon based, peptide or protein based NPs, and organic NPs have also been reported in NP based studies.^29,30^

#### 2.1.2. Size of nanoparticle

Among the numerous parameters involved during the complex interaction of NPs with the bulk biological environment, the size plays a major role that ranges from the determination of the dynamics of protein corona formation to the delivery of NPs to the target tissues.^31,32^ Even though there are continuous size ranges, and the size classification can result specific to their application, generally NPs can be considered as small if their size is between 1 nm and 30 nm, medium in the size range 30 nm to 70 nm, and large NPs within 70 nm to 100 nm range.^33^ Given the heterogeneous and interconnected nature of NPs' interactions with the cellular milieu, their size ultimately determines the amount of molecules adsorbed onto their surface. First, the particle sizes with their surface to volume ratio determine the characteristics of the PC, the kinetics of its hardening that in turn determine the quantity of proteins adsorbed.^34^ Secondly, the size of NPs influences the thermodynamics of protein adsorption such as enthalpy and entropy changes, that in turn control the adsorption effect of NPs.^35,36^ Thirdly, NPs size determines the morphology of the corona^27^ and affects the conformation of proteins adsorbed on the NP surface.^37,38^ Other characteristics, such as heterogeneous nature and relative density of adsorbed PC, are also dependent on the NP size.

Size of NP seems to determine also the binding affinity that critically governs the absorption and desorption equilibrium of protein corona.^39^ As demonstrated from different NPs such as solid-lipid NPs, AgNPs, and AuNPs, a weaker affinity with the protein was observed when the NPs are small sized.^40,41^ On the other hand, the larger the size of NPs, the greater the interaction forces are needed for gathering; large sized NPs tend to have larger potential interfaces.^36,42^ Wang *et al.* also employed a combination of versatile techniques and demonstrated the effect of size of solid lipid nanoparticles that ranges from 120 to 480 nm at pH 6.0 and 7.4 onto the bovine serum albumin and revealed reduced protein adsorption on small particle size and hydrophilic surface of nanoparticle.^43^

Numerous studies, *in vitro and in vivo,* on the interaction of nanomaterials with cells demonstrated the differential roles of adsorbed proteins as a function of the surface of NPs. For example, smaller sized NPs (size <70 nm) showed lower affinity to Human Serum Albumin (HSA) as compared to larger sized NPs (size around 200 nm).^44^ Similar findings were also reported in the cases of metal NPs,^45^ silica NPs,^46^ polymeric NPs^47^ and polystyrene NPs.^48^

NP size has also a direct influence during extravasation of NP in the circulation and cellular uptake. Small NPs generally cross capillary walls more easily than large NPs.^49,50^ In line with this, marked differences on cellular uptake mechanism have been observed in endothelial cells for different sized NPs. As a result, while 50 nm NPs were primarily internalized by caveolin-mediated endocytosis, the internalization of 70 nm NPs occurred through clathrin-mediated endocytosis.^51^ These facts highlight the impacts of the size of NPs on the phagocytosis of macrophages and its consequences results on inflammatory and humoral response.^52^

Overall, apart from the formation of protein layer during the biodistribution, NPs will face other biological and physical barriers. Thus, to overcome the barriers, controlling the NP size is one of the widely practiced mechanism during nanoparticle design.^53^

#### 2.1.3. Shape of nanoparticle

Given the advancements of modern synthesis techniques, NPs can be engineered and designed into variety of shapes. Among these, spherical shaped NPs are commonly used in most protein corona research literature. Others such as rod, sheet, tube diamond, star, cube shaped NPs *etc.* have also been reported.^29,54^ Nanoparticles shape is one physical parameter which exerted a significant effect on protein corona formation with a resultant change in the biological properties or biodistribution of the nanoparticles.^55^ The change in shape of nanoparticles also influence protein corona architecture.^56,57^ Indeed, as demonstrated for gold nanoparticles, PC formation and aggregation behaviors of the AuNPs were largely influenced by their morphologies that in turn affected the structures and functions of adsorbed fibrinogen (FIB) and trypsin (Try).^58^ Similarly, adsorption behavior of immunoglobulins and albumin proteins from both plasma and serum was correlated with the shape of nanoparticle.^59^

#### 2.1.4. Surface charge

The surface charge of NPs is another factor that regulates the composition of protein corona (PC), the NP–PC binding affinity, and the proteins structural changes, which ultimately affects the interaction of proteins with each other and the functions of NPs in biological systems.^60–63^ A study on plain carboxyl-functionalized and amino-functionalized polystyrene nanoparticles showed that the surface chemistry of the nanomaterial determines the protein composition of the ∼15 nm hard corona.^64^ It has been also evidenced that even a small initial difference in the surface of nanoparticle could lead to significant change in the protein adsorption and thereby, in different protein layers. From their analysis of protein corona formation on mesoporous silica nanoparticles, Fener-Garcia *et al.* clearly demonstrated surface dependent evolution of heterogeneity in protein absorption over time.^65^ From serum albumin and lysozyme coated polystyrene nanoplastics of different nanometer sizes and charges, regardless of the protein type, the soft corona complexes adopted a structure where the nanoplastics are surrounded by a loose protein layer and hard corona complexes formed fractal-like aggregates.^66^

Positively charged NPs consistently adsorb more proteins than NPs bearing negative surfaces independently from the type of material used. However, increasing the negative surface charge density on nanoparticles can also increase the mass of adsorbed protein.^67^ The adsorbed proteins retain their folded structures on anionic or neutral surfaces, while they become unfolded on cationic surfaces, which more predominantly occurs for larger particles with lower surface curvature.^68^ Remarkably, the work from Oberländer and his colleagues demonstrated that even at fixed temperature and plasma concentration, the surface charge influence the protein corona composition of polystyrene nanoparticles.^69^

#### 2.1.5. Hydrophobicity

Several studies have revealed that highly hydrophobic nanoparticles can absorb larger quantities of protein compared to the hydrophilic ones.^67,70^ Hydrophobicity is examined as the determinant of the protein adsorption onto NP surface and circulation time,^71,72^ implying that the hydrophilic and hydrophobic feature of NPs should be considered in the methodology design. For example, Bewersdorff *et al.* observed differences in corona formation with varying hydrophobicity of Nanogels (NGs). With respect to hydrophilic NGs they observed a significant increment in the hydrodynamic diameters of hydrophobic NGs suggesting an increased protein adsorption.^73^ Due to their higher hydrophobicity riboflavin-coated superparamagnetic iron oxide nanoparticles adsorbed more serum proteins than the bare one, although both showed similar sizes and zeta potentials.^74^ On the other hand, protein adsorption onto NPs can be suppressed by coating their surfaces with different molecules like zwitterionic, PEGylated or carbohydrate moieties, which produce extremely hydrophilic NPs.^75,76^ Subsequently, without proteins present on their surfaces, these hydrophilic NPs can evade clearance from the immune system and for this reason they are called “stealth” NPs.

#### 2.1.6. Surface chirality

The interaction between the NPs chiral surface and the bioactive molecules determines the surface energy of the NP, thereby influencing their bio-distribution and cellular uptake efficacies.^77^ The chiral surface of NPs governs the orientation and conformation of PC, influences protein adsorption and mediates the interaction with specific categories of proteins.^78,79^ Stereo-selective interactions, due to chiral subunits, influence the protein adsorption dynamics. For example, a study on chiral gold NPs demonstrated distinct cell uptake and tissue accumulation *in vivo* with diverse protein composition, including lipoproteins, complements, and acute phase proteins.^21^ Similarly, the chiral molecule determines the amount of internalized molecules, as demonstrated from poly-l-lysine and poly-d-lysine coated periodic mesoporous organosilica, which results in cells to internalize more poly-l-lysine than poly-d-lysine.^80^

It is of interest to note that recent research introduces a robust, thiol-independent ligand engineering strategy to precisely modulate protein corona formation on nanoparticles across different biological compartments. This approach enables researchers to move beyond conventional, often unstable thiol-based attachments, thereby allowing for the deliberate selection of protein adsorption profiles both in the bloodstream and within the intracellular environment.^81^

### 2.2. Influence of biological environment on PC formation

Although the driving forces and mechanisms of the conformational changes in proteins adsorbed on NP surface remain greatly unknown, it is widely recognized that both interactions, protein–NP and between neighboring proteins on NP surface, can influence protein adsorption behavior and conformational changes.^82,83^ For these reasons, the crowded environment inside cells ultimately determines the destination of nanoparticles. The type of protein source and incubation environment, among others, influence the protein corona formation and protein identification.^84,85^ In line with this, in this subsection we highlight the impact of temperature, pH, source of biological fluids and other related factors on PC formation.

#### 2.2.1. Temperature

The temperature at which the protein corona is formed affects its composition on each NPs surface,^86^ in terms of type and amounts of proteins,^87^ and influences also the binding affinity of proteins in the NPs hard corona.^88,89^ In fact, a pronounced effect of temperature was observed for magnetic NPs between 5 to 45 °C.^90^

The absorption of proteins on NPs could be increased or decreased by temperature variation, and this could be due to the change in protein conformation. For instance, while the proteins in their native conformation have specific characteristics in terms of hydrophobicity, specific surface charge, and exposed residues, conformational changes, such as partial or complete unfolding, can expose new hydrophobic regions that could subsequently lead to changes in the amount of protein absorbed on the NPs surface.^91,92^ Interestingly, Prawatborisut *et al.* observed that at physiological temperature (37 °C) the NPs were enriched with apolipoprotein A1 and apolipoprotein E while 25 °C favored apolipoprotein J (clusterin) absorption.^24^

Moreover, the effect of the incubation temperature on the corona composition can vary based on the concentration of the protein sample.

The significance of working in temperature controlled environment can also be explained in terms of the role of temperature in cellular processes. The reason behind this is related to endocytosis processes that are naturally temperature-dependent.^93,94^ In fact, temperature-dependent endocytosis of single-walled carbon nanotubes was observed at various temperature windows.^94^

At increasing coating temperature (4 °C, 25 °C, and 37 °C), Oberländer *et al.* observed decreased NPs uptake by cancer and endothelial cells. In their analysis, Apolipoproteins, clusterin and ApoE proteins were enriched across different NPs, and the composition of the PC was generally different at low temperatures (4 °C) and at physiological temperatures (37 °C).^24^

#### 2.2.2. pH

Since the pH of biological microenvironment was found markedly different in diseased and normal conditions, a detailed evaluation of corona formation in different pH conditions is of paramount importance to understand the corona dynamics. So, the model cells lines used in the experimental settings should represent the pH of the microenvironment where the NPs are targeted.

Indeed, external factors like pH of the medium can influence the conformation and charge of the proteins on NPs surface,^95^ and thereby affects their interaction.^96^ Without neglecting the impact of other factors on corona composition, a study conducted on bovine serum albumin and gold NPs reports few adsorbed proteins at pH 7.4 with unaffected secondary structure of the protein while aggregation of proteins on NPs with altered folding of the secondary structure was observed at pH 4.0.^97^

In this regard, the secondary structure content of each protein can determine the inherent stability of the protein, which in turn contributes to the conformational changes upon NP interaction.^96^ For example, the human serum albumin (HSA), one of the most abundant protein in blood plasma and component of protein corona, is known to undergo pH-dependent conformational and functional changes.^98^ The structure of HSA was also demonstrated influenced by PEG-OMe, PEG-COOH, PEG-NH2, and glycan functionalized AuNP with the observation of different degree of corona formation at acid, neutral and basic pH.^99^

Besides, the intensity of interaction between different NPs and the protein corona depends on the pH and ionic strength. For instance, the molecular weight and isoelectric point of each specific protein type, pH, and ionic strength were shown to determine the dispersion or agglomeration behavior of gold nanoparticles in protein solutions.^100,101^ In line with this, gold NPs were found to be stable in neutral and alkaline solutions while aggregation took place at lower pH.^102^ Importantly, the effect of ionic strength on NP aggregation can vary with the NP size.^103^ As such, from the commonly studied model system of silica NPs and their interaction with lysosome proteins, different patterns of morphology have been shown that depend from NP size and pH. As a result, a decrease in pH enhanced electrostatic interaction increasing the adsorption coefficient on NPs.^95^

Over all, the composition of protein corona depends on the strength of interaction at the nano-bio interface^104^ since different pH can maintain different interaction forces. For example, the interaction between solid lipid nanoparticle and bovine serum albumin is maintained principally by van der Waals forces and hydrogen bonding at pH 7.4 while at pH 6.0 electrostatic attractions are favored.^40^

#### 2.2.3. Source of biological fluid and related factors

Protein corona formation is a dynamic process, which makes difficult to fully understand the influence of blood proteins on the structure assembly and dynamics at the biomolecule–nanoparticle interface. This multi facet interaction relies on the amount of protein, and their interaction time controls adsorption process, whereas, desorption from NP surface depends on binding energy of protein-nanomaterial complex.^19^ Numerous *in vivo* and *ex vivo* studies demonstrated the effect of source of sample, plasma concentrations, incubation time, animal sex on the protein corona composition in different nanoparticle systems.^23,24,88^ Thus, changes in type and composition of biofluid can lead to differences in the protein corona composition on NPs surface.^84,105^ For instance, in the dynamic flow study of lipid NPs circulating fetal bovine serum in human cervical cancer (HeLa) and human breast adenocarcinoma (MCF7) cell lines, the PC composition has been shown dependent on both exposure time and NP's surface with a notable effect of shear force on PC.^106^

From the reports of literature, there are disagreements between PC formed on NPs in *in vitro* and *in vivo* experiments, that makes a challenge to do comparisons. Researchers hypothesized that varied results may be partly due to the vessel structure and its impact on fluid dynamics. Therefore, significant variation of *in vivo* corona formation with respect to *in vitro* may be observed in terms of both variety and quantity of proteins.^107^ Additionally, loosely attached protein corona on NPs may strip off because of fluid sheath force, as demonstrated in fluid flow mimicking biological vessels.^108^ Collectively, evidence suggests that injected NPs traverse vascular networks, and the vascular features by themselves can impact the protein corona composition.

Another finding that could explain the complexity of the bio-nano interface dynamics is the finding from Nandakumar and his researcher group who have identified 58 proteins that are unique to the Aβ_1–42_ samples and 31 proteins unique to the Aβ_1–40_ samples in human blood plasma. All of these identified proteins are fibrillar coronae significant in complement activation, inflammation, and protein metabolic pathways.^109^

As demonstrated from different studies, another factor that influence the protein corona composition is the incubation time: different time point incubation enables to resolve or characterize the evolution of proteins.^110^

In general, the dynamics of intracellular release or exchange of the blood protein corona from nanomaterials following their cellular internalization, and the biological footprints of the nanoparticle–protein corona traversing cellular compartments, are diverse and the mechanism seems less understood. On the other hand, precise manipulation of interaction modes of functional plasma proteins on the nano-surface could provide corona-mediated targeting for specific drug delivery.

### 2.3. Physicochemical properties of nanoparticles: implication on neurodegenerative diseases

In this subsection, we introduce NPs and their interactions with amyloidogenic proteins, and the challenges that parameter variability poses to the reproducibility of NP-related reports. Details on the effect of NPs in either accelerating or inhibiting the pathological hallmarks of neurodegenerative diseases and PC-amyloid protein interaction is discussed in Section 4.

Among the various properties of NPs investigated, a plethora of research has shown that the NP surface acts as a heterogeneous catalyst that induces a conformational switch in amyloidogenic proteins. Consequently, surface charge of NPs promotes early aggregation of amyloidogenic peptides and accelerate oligomer formation.^111^ For instance, TiO_2_–NH_2_ NPs with a positive surface charge showed higher tendency to adsorb the Aβ42 peptide, thereby enhancing the capacity of Aβ42 oligomer aggregation.^112^ In the case of α-synuclein, its amphiphilic N-terminal or acidic C-terminal domains provide alternative interactions with positively or negatively charged NPs. Nonetheless, both negatively and positively charged nanoparticles have been shown to either inhibit or accelerate α-synuclein fibrillation.^113^

In this regard, it is of interest to mention that targeted application of engineered nano-approaches leverages precise molecular design strategies, including nanobodies, nanozymes, and advanced engineered NPs.^114^ As such, advanced engineered NPs hold promises for precision medicine as a therapeutic or diagnostic device. For instance, design advancements in the shape of polymer NPs, which are useful for treating neurological diseases involving inflammation,^115^ and the surface modification of inorganic NPs, applicable as both therapeutics for neurological disorders^116^ and diagnostics for Alzheimer's disease,^117^ are notable examples of successful design approaches to meet solubility, administration and biodistribution requirements. These findings underscore that the surface charge of NPs is central to neurodegenerative diseases intervention.

However, despite these advancements and opportunities, research on NPs in Alzheimer's and Parkinson's is limited by short-term experimental studies that fail to capture the long-term, real-world effects of chronic exposure on neurodegeneration. Moreover, the reports in the nanomedicine field did not commonly consider the effect of the protein corona on fibrillogenesis of amyloidogenic proteins.^114^

Indeed, the variability in nanoparticle physicochemical characteristics, including surface chemistry, charge, size distribution, agglomeration state, hydrophobicity, hydrophilicity and PC formation introduces significant heterogeneity across studies. Consequently, this challenges the reproducibility of reports in disease-related research. Furthermore, the clinical use of nanotherapeutics can be hindered by poor BBB penetration, instability, and safety concerns, such as off-target effects and immune reactions.^114^

## 3 Research methodology approaches for preparation and characterization of the protein corona

**Fig. 2:**
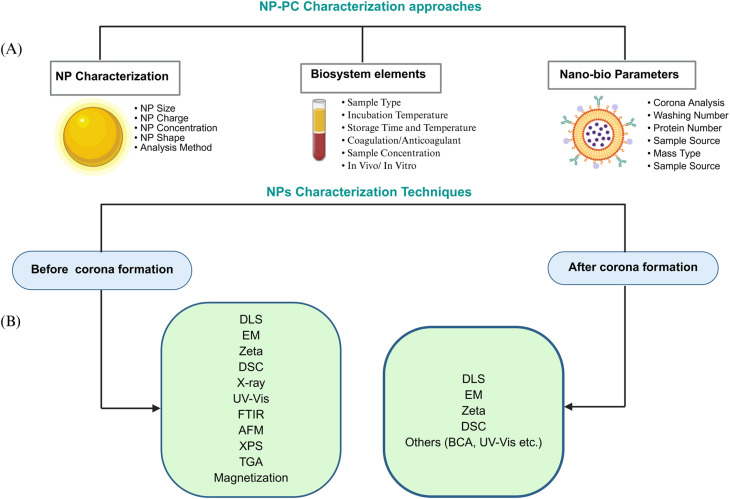
Overview of NPs characterization approaches and techniques.^86^ (A) The formation of the protein corona on NPs and its robust characterization mainly require parameters from NPs characterization, nano-bio parameters, and biosystem elements listed in (A), but not limited to those. (B) Characterization of NPs before and after corona formation. Among several techniques, DLS, EM, and zeta potential are the major methods used for characterizing NPs. These methods are also employed to investigate the protein corona on NPs and include other techniques as well, generally focusing on size changes, morphological alterations, NP–protein interactions, protein conformation changes, and other characteristics.

In the field of nanotechnology a comprehensive understanding of the interaction of NPs with biological systems is critical in designing and developing safe and efficient clinical diagnostic and therapeutic applications with approaches reflective of the vast physiochemical complexity of nanoparticle-based agents.^6,29^ Despite myriads of published studies, application of various analytical techniques and a wide range of experimental conditions in the protein corona research, there is no standard protocol on methodologies to provide the most precise and comprehensive characterization of the protein corona. Furthermore, it is highly possible that non-standard methodology could induce misinterpretation and poor outcomes. Consequently, this poses a challenge in the reproducibility and reliability of methodologies in nanomedicine. Therefore, considering each nanoparticle formulation, there is a need to develop standard analytical approaches and characterization techniques for robust and precise investigation of parameters of the nanoparticle, bio-system and the nanoparticle-protein corona interaction surfaces (Fig. 2).

Herein, in the following subsections, we highlighted the experimental facts and challenges regarding methodology and characterization of the protein corona. We also introduce field expert's proposed strategies that can be applied to minimize the shielding effect of protein corona.

### 3.1. Preparation of protein corona (preparation, washing media, and isolation *etc.*)

The successful translation of drug delivery vehicles into clinics relies on the biological microenvironment, robust characterization of nanomaterials and accurate methodological approaches to study the formation and subsequent analysis of the protein corona.

In general, the composition of protein corona and surface interactions with nanoparticles are being studied in pure protein solutions,^118^ in complex protein solutions, such as blood serum,^119,120^ in cell lysates,^121^ and in living cells.^122^ As compared to serum, *in vitro* model based studies demonstrate significant differences in protein corona composition.^123^ However, *in vivo* corona formation is more complex and not static (*i.e.* it changes over time) thereby acquiring new tissue- or cell-specific proteins from the local environment.^124^ In this regard, at the initial stage, rigorous quality control of the biological fluids such as human sources (serum/plasma), bovine derived biological materials and other sources must be undertaken for the best possible precise protein corona analysis. Moreover, as preparation and collection of the biological fluids can affect the accuracy of the corona analysis, care must be taken into consideration on the source of sample, sample history, storage medium, sex, age and other critical factors.^84,125,126^ For instance, notable differences of protein concentration on the same nanoparticles were observed among osteoarthritis patient synovial fluid and fetal calf serum protein sources.^127^ Similarly, *in vitro* study of polystyrene nanoparticles incubated in fetal bovine serum, human serum, human citrate and heparin plasma, clearly demonstrated the influence of protein source and cell type on nanoparticle uptake. As a result, macrophage cell line showed more uptake for nanoparticles incubated in human heparin plasma, whereas the HeLa cells had greater uptake for those incubated in human citrate plasma.^128^

Based on the nature of each corona type, mixed corona, soft corona and hard corona, the number of washing steps has different effects. Different washing numbers in different isolation methods such as centrifugation or magnetic retrieval were reported to yield different protein concentration in mass spectrometric proteomic analysis.^129^ As such, at least three washing steps were recommended to ensure the effective removal of unbound protein from the original biological fluid.^130,131^ Meanwhile, washing media influence the composition of the hard corona of different nanocarrier systems and, thereby, affects the protein stability and cellular uptake behavior.^132^ Hossen *et al.* observed from 2 to 22 fold variation in PCs composition on 20 nm gold-NPs (AuNPs) incubated with urea lysates as compared to radioimmunoprecipitation assay (RIPA) lysates,^133^ suggesting the importance of pre-processing conditions to modulate the PC composition. Another important factor is the type of cell culture media. For example, apart from differences in the composition of hard protein corona, researchers observed different degree of dispersion stability of bare and hard corona-coated single layer and multiple layer graphene oxide materials in ultrapure water, DMEM medium, and DMEM with 10% of fetal bovine serum,^72,134,135^ suggesting the effects of culture media on protein corona–nanoparticle interaction.

The percentage of incubated protein and selective sequential pre-formation of protein complexes prior to incubation can also alter the biological behavior of nanoparticles.^136,137^ The formation of single layer protein can affect the aggregation of proteins. The presence of a pre-incubated layer of fibrinogen around the nanoparticles, before a protein corona is formed in bovine serum, showed significant increment in the cellular uptake.^138^ Another factor that should not be ignored at this stage is the choice of the incubation temperature since the stability of the protein changes as function of the temperature, and thereby impacts the outcomes.^69^

Investigation of the hard protein corona is the starting phase to scrutinize the biological behavior of NPs. Therefore, the choice of appropriate hard protein corona isolation methods is quite important. It is well known that centrifugation is the most commonly used standard method to isolate NPs from a biological matrix,^29^ but, for deeper understanding of all corona types, experts in the field recommended more gentle means of retrieval and then, quantitative proteomic analyses to provide qualitative characterization of protein corona.^139^ In addition to the existing magnetic separation method (MagSep), a multi-step centrifugation method (MSCM) showed better reproducibility. The MSCM for single-domain magnetic NPs, applied to iron oxide NPs in interaction with human blood and lymph serum, showed the different hard protein coronas content higher than when the MagSep is applied.^140^

In centrifugation-based protein-separation techniques the effect and suitability of different eluents should be also considered. For example, different protein denaturants like sodium dodecyl sulfate, dithiothreitol, and urea significantly contributes for efficient desorption of PC on gold nanoparticles and silica nanoparticles.^141^ In phenol red, penicillin-streptomycin, l-glutamine, and β-mercaptoethanol supplemented cell culture media, the NP-protein corona took more time to form with altered density and composition.^142^

Methodological advancements and availability of suitable techniques can help the comprehension of the differential expression of proteins under physiological conditions. For example, different groups of researchers utilized high-performance liquid chromatography coupled with electrospray ionization with ion trap mass analyzer (HPLC/ESI-Orbitrap) and have identified hundreds of differentially expressed proteins. Using different software search engines like MASCOT and gene ontology they also characterized proteins functional behavior and interactions based on their regularity, locality, molecular functionality and molecular masses.^143,144^ Application of specific X-ray photoelectron spectroscopy with other protein separation techniques enabled to discriminate stably adsorbed coronas from weakly adsorbed coronas from functionalized and unfunctionalized nanoparticles.^145^ In another study, Szekeres *et al.* applied combined approach of reversed phase high-performance liquid chromatography coupled with quadrupole-time-of-flight mass spectrometer using electrospray ionization, and were able to identify the hard corona proteins of citrate-stabilized gold nanoparticles in MCF-7 cells and J774 macrophage cells.^146^

Similar to protein corona in the circulation, around the extracellular vesicles intracellular protein corona^147,148^ can be formed on nanoparticles when transported inside the cells. Therefore, it is essential to back-track the transport pathways of nanoparticles. For this case, application of high-performance liquid chromatography-tandem mass spectrometry-based proteomics with dual-filtration strategy enables research groups to identify specific intracellular corona proteins.^149^

Another key point in PC formation is the issue of homogeneity or heterogeneity. To face this problem, analytical techniques such as fast screening mechanism can be applied before quantitative analysis of the protein corona composition. In this regard, Magnetic levitation (MagLev) is the most suitable tool and is becoming a promising technique to separate corona coated NPs.^150^ Similarly, given the fact that recovering the protein corona from the nanoparticle surface is necessary for characterization, the gold standard nano-LC-MS is being used for this specific purpose. Capillary electrophoresis with electrospray ionization mass spectrometry and on-particle tryptic digestion methods also showed higher reproducibility.^151^

**Fig. 3:**
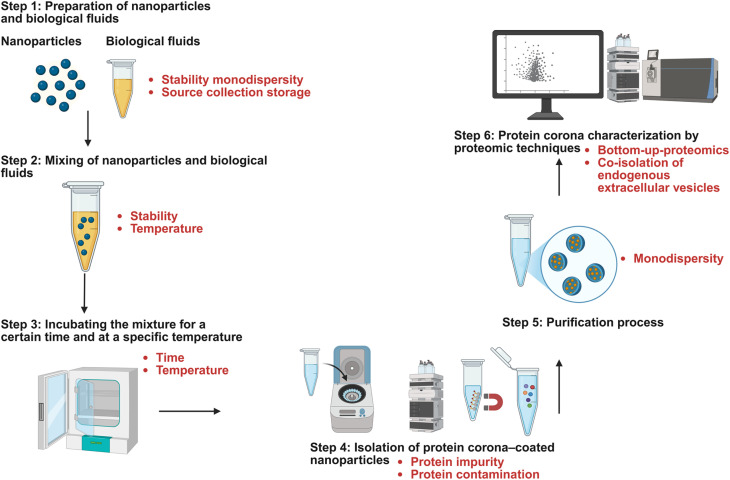
General process for the preparation of protein corona. The preparation of a protein corona for *in vitro* or *ex vivo* analysis follows a standardized workflow involving: (1) preparation: preparation of NPs and biological fluids (*in vitro* or *ex vivo*). (2 and 3) Mixing and incubation: mixing of NPs and biological fluid under specific time and temperature conditions. (4) Isolation: separation of the protein corona coated NPs using five primary methods: centrifugation-based, gradient centrifugation, size exclusion chromatography, magnetic separation, field-flow fractionation. (5) Purification: removal of the unbound proteins and impurities. (6) Characterization: identification and quantification of the corona using proteomic techniques. The text highlighted in red identifies methodological patterns known to introduce errors into PC proteomics results.

Nonetheless, while traditional analytical methods like gel electrophoresis, mass spectrometry, and surface plasmon resonance provide structural insights, they often fail to capture the dynamic, heterogeneous protein-nanoparticle interactions occurring in real-time *in vivo* conditions. Indeed, the co-isolation of endogenous impurity nanoparticles, including extracellular vesicles (EVs) is a significant challenge in the final proteomics analysis.^152^ Standard isolation techniques like centrifugation or magnetic separation often pull down EVs and other endogenous biological nanoparticles from the biofluid, thereby introducing highly contaminated corona profile in the final proteomics stage (Fig. 3). This leads to false positive outcomes in the number of identified proteins and wrong conclusion on the binding affinity of proteins to NPs. Interestingly, a recent comparative study of standard pooled human plasma and EV-depleted plasma (using the immunoaffinity MACSPlex multiplex bead platform) revealed that the removal of endogenous EVs led to a substantial 60–75% drop in proteins identified on polystyrene NPs and a 45–50% drop on magnetic beads.^152^ These findings highlight that moving beyond the mere observation of a nanoparticle's ‘biological identity’ to actively defining its true composition is a necessary evolution for the field.

### 3.2. Overview of common and emerging techniques for characterization of PC–NP complex

Understanding the composition and kinetics of the PC at the molecular level is of considerable importance for controlling NP interaction with cells. Altering the bulk physico-chemical properties of NPs is the widely practiced approach to target specific protein subsets. Even though, in most cases its effect on protein adsorption, either to increase or decrease it, depends on the NP system, a general relationship between surface chemistry and protein adsorption that applies to any NP system has not yet been established.

For the study of PC formation in terms of kinetics, researchers suggested three phases model characterization. In the first phase, proteins that are irreversibly and directly bound to the nanoparticle surface are categorized. In the second and third phases, irreversibly bound proteins interacting with preadsorbed proteins, and reversibly bound soft protein corona proteins, can be characterized.^153–155^ As such, classical *in situ* characterization methods, including dynamic light scattering (DLS), transmission electron microscopy (TEM), fluorescence correlation spectroscopy (FCS), circular dichroism (CD) spectroscopy, and isothermal titration calorimetry (ITC) provide critical insights into the size and morphology of NP–protein complexes, the stoichiometry and binding kinetics of their interactions, and the conformational transitions that proteins undergo upon surface adsorption.^156,157^ A concise summary of the most common and currently emerging tools and their application is provided in Table 1.

**Table 1:** Summary of most common and recent techniques for examining and characterizing PC–NP complex

Method	Application	Advantage	Limitation/challenge	Ref.
DLS (dynamic light scattering) and zeta potential	• Routine screening of PC formation and colloidal stability	• Fast, non-destructive	• Cannot identify specific protein types	86
		• Standard for measuring hydrodynamic size and charge shifts	• Sensitive to polydispersity	
SAXS (small-angle X-ray scattering)	• Emerging as most suitable tool for *in situ* corona analysis	• Better for resolution of polydispersity	• Difficultly to resolve the low-contrast, thin protein corona	166
	• Resolves interactions at the nano–bio interface	• Suitable for very concentrated samples	• Limited to discriminate protein layer from background (buffer solution)	
		• Analyzes the entire population of particles in solution	• Limited for heterogeneous or highly complex systems	
Photocatalytic proximity labeling technology in nanoparticles (nano-PPL)	• Emerging tool	• Captures soft corona in real-time with 5 seconds of precision	• Requires photosensitive probes like chlorin e6 catalyst and biotin-phenol	167
	• High-resolution dynamic studies of the soft and hard corona evolution	• Rapid and precise labeling of corona proteins *in situ*		
cIEF-MS/MS (capillary isoelectric focusing-MS/MS) (top-down)	• Advanced proteomic fingerprinting for biomarker discovery	• High-throughput	• Requires complex instrumentation and data processing	168 and 169
		• Preserves proteoforms and post-translational modifications	• Challenging to identify large proteoforms (>30 kDa) from complex samples	
LC-MS/MS (bottom-up)	• Comprehensive identification and quantification protein on NPs	• High sensitivity	• Poor reproducibility	170
		• Identifies protein identity	• Relies on ensemble-averaged measurements	
		• Easier to separate and ionize	• Loos of soft protein corona	
			• Peptide level uncertainty	
			• Limited to size range	
AFM (atomic force microscopy)	• Visualizing structural changes and orientation of adsorbed proteins	• Nanoscale visual resolution of single-molecule bioconjugation	• Low throughput; sample preparation (drying) may alter the corona structure	156
		• High resolution, low invasiveness, and minimal destructiveness	• Limited in discerning formation of hard or soft corona	
DCS (differential centrifugal sedimentation)	• Characterizing complex, multi-layered coronas in heterogeneous samples	• Higher resolution than DLS for multi-modal size distributions	• Requires precise knowledge of particle and medium density	171
		• Suitable for monitoring subtle changes in dispersion stability resulting from thin surface coatings	• Limited for complexes <5 nm in size	

Current hypotheses underscore the pivotal role of charge screening in mediating corona formation. The rationale is that the protein corona development is governed by collective interactions at the nano-bio interface driven by a complex interplay of forces, including hydrogen bonding, steric hindrance, hydrophobic interaction, van der Waals forces, electrostatic and entropic contributions. This has been demonstrated in different materials such as in Ti_3_C_2_T_*x*_ nanosheets,^158^ DNA-functionalized single-walled carbon nanotubes,^159^ monodisperse silica nanoparticles,^160^ N-acetyl-l-cysteine-capped CdTe quantum dots (QDs),^161^ Zn doped cadmium-based quantum dots^162^ and others.^163,164^

Super-resolution microscopy (SRM) is another powerful and growing tool that can be applied in a broad range of nanomaterials under physiological conditions. However, plasmonic substances, such as gold, may quench fluorescence and special considerations should be taken while working with such type of materials. Beyond these challenges, strict labeling requirements, potential artifacts, and the need for complex instrumentation and technical expertise render SRM less applicable to the current protein corona research.^165^ Weiss *et al.* employed a combination of confocal laser scanning microscopy with microfluidics to trace the time-evolution of protein corona formation *in situ*. As a result, during the particle-protein interactions on low-fouling zwitterionic-coated particles they observed only a soft protein corona formation onto silica microparticles.^155^

Characterization of nanoparticles before and after exposure to sample (serum/plasma) is a fundamental prerequisite for precise protein corona analysis. Therefore, controlling the particle size is the most feasible method during NP design. To this aim, the size, morphology, hydrodynamic size, and zeta potential are extensively studied by transmission electron microscopy and dynamic light scattering.^29,172^ In a comprehensive research work done by Hajipour and co-workers,^29^ DLS was described as the most conventional method that is used to determine the hydrodynamic size distribution of NPs before and after adsorption of protein corona. However, it has been shown that most studies do not report the standard deviation for DLS or charge measurements, highlighting the challenge of assessing the reproducibility of data acquisition for coronated NPs.^29^ Reasons to choose DLS over others could be due to its sensitivity to soft biomolecules leading to a better suitability to study size change of NPs as spherical nanoparticles.^173,174^

FCS and fluorescence cross-correlation spectroscopy (FCCS) are also sensitive techniques that measure fluorescence intensity fluctuations of single molecules inside a femtoliter confocal volume. Protein corona stability *in situ* in biological matrix was studied using these techniques.^175–177^ Single-molecule sensitivity allows FCS to be performed with very low sample concentrations and volumes. Furthermore, using fluorescent labeling in multi-color FCCS experiments provides a distinct advantage over DLS.^178^ A shared limitation of both FCS and DLS that warrants specific attention is an overrepresentation of large particles in the data.

As extensively reviewed by Fu *et al.*, TEM is ideal for visualizing the morphology and detailed microstructures of NPs. However, the use of non-specific contrast agents may potentially disturb the proteins' secondary structure, leading to artifacts and possibly misleading conclusions.^156^

As demonstrated from the biochemical and biophysical characterization, the interaction of ultrasmall GNP (usGNP) with proteins like chymotrypsin, trypsin, thrombin, serum albumin, cytochrome c, and factor XII, permanently altered protein function in time dependent manner. The effect of both short-(10 min) and long- (24 h) term interactions yield short-lived complexes, indicating no time-dependent “hardening” of the interactions at the binding interface as usually seen with large GNPs.^179^

Beyond the biophysical techniques highlighted here, qualitative and quantitative approaches such as western blot and Bradford assay, SDS-PAGE and LC-MS/MS can be used to evaluate the impact of surface end group identity, matrix polymer hydrophobicity, molecular weight of distinct serum and cellular proteins, and NP formulation agents like Poly-lactic co-glycolic acid on the protein corona composition.^72,180^

As discussed earlier, one significant challenge in traditional methods, such as mass spectrometry, is the loss of weakly bound proteins during sample preparation. Consequently, alternative and more robust strategies are required to capture the full protein corona profile. In this regard, NMR spectroscopy is a promising tool for studying PC-NP interactions in their native, liquid-state environment without the need for destructive separation. For instance, from a study on interaction of polystyrene nanoplastics with human ubiquitin protein at atomic resolution, researchers were able to discern protein corona formation, conformational changes in ubiquitin and protein induced coalescence of nanoplastics.^181^

Moreover, together with experimental procedures, molecular dynamics simulations can be a complementary tool to deeply characterize at atomic level the interaction phenomena occurring in the protein corona formation and how the physicochemical properties of NPs can modulate them. Indeed, coarse-grained and mesoscopic simulations allowed to study bigger complexes by-passing the limitation of computing power.^182^

### 3.3. Emerging advances, current gaps, and strategies towards NP-protein corona methodological standardization

The vast majority of protein corona literature to date has used the traditional bottom-up proteomics (BUP) approach, where proteins are digested into small peptides before being analyzed by the mass spectrometer.^86^ However, BUP fails to distinguish between different proteoforms, leading to a loss of critical post-translational modification data and an inability to predict the specific biological interactions of NPs.^183^ This underscores the urgent need for method integration. Application of top-down proteomics (TDP) enables direct analysis of intact proteins and provides a more accurate map of the active corona.^184^ Thus, current research is pushing for TDP. It is noteworthy that the successful integration of mass spectrometry-based TDP and BUP advances proteoform-level analysis of the protein corona leading to identification of specific proteoforms.^185^ Remarkably, using TDP, Morteza and his team identified 3505 proteoforms from 344 genes in human plasma samples. This is a 4-fold increase over previous polystyrene nanoparticles studies and currently stands as the largest proteoform dataset ever reported for a protein corona. Similarly, the BUP analysis identified 4570 protein groups and over 45 000 peptides. Notably, by combining these massive datasets, the researchers improved the characterization quality of 35% of all identified proteoforms that showed mass shifts.^185^

There is also a significant development towards *in situ* and high-throughput techniques that capture the soft corona, which traditional centrifugation-based methods often lose.^156^ Emerging automated platforms and microfluidic technologies now enable the high-throughput screening of hundreds of nanoparticle formulations within the complex biological milieu. Microfluidic devices can bridge the gap between *in vitro* and clinical studies by precisely controlling microenvironments (geometry, pH, and temperature). Thus, application of microfluidic platforms for *in vitro* NP evaluation is undoubtedly promising.^186^

Moreover, in the protein corona research, the development of machine learning models can also contribute to predicting protein corona compositions and the consequent biological impacts.^187,188^ As an example, Sengottiyan *et al.* developed quantitative structure–property relationship (QSPR) (nano-QSPR models) specifically targeted to calculate zeta potential (*ζ*), one of the crucial parameter determined by the complex interaction of corona and NP, with the aim to quantitatively correlate it with the coating of NPs, and their PC fingerprints.^189^ This study employed corona-derived descriptors to effectively demonstrate how the protein corona re-codes a NP's surface charge. This approach represents a biological identity model that offers a distinct predictive advantage in calculating the zeta potential of NPs within the biological milieu, positioning the work as a cornerstone for global standardization and modeling efforts.^189^

However, despite these emerging promising advances, there is still a major gap regarding clinical translation and industrial scalability of NPs. These necessitate a scientific consensus and community initiatives to advance the nanomedicine field. For instance, there is a significant lack of data on how patient-specific factors, including age, sex, disease state like neurodegenerative, diabetes or inflammation alter the PC. This personalized protein corona tailored for personalized medicine is an emerging concept that needs a shift from standardized serum samples to patient specific NP-PC analysis.^190^ Moreover, the vast amount of descriptive data on NP-PC interactions can be leveraged by robust machine learning frameworks to accurately predict the corona fingerprint of a novel synthetic particle prior to its synthesis. Indeed, the role of an *in silico* based predictive modeling approach has been ignored to date. Another major critical gap lies in the analysis of the *in vivo* protein corona. Despite numerous bottlenecks regarding an *in vivo* corona analysis, the successful translation of nanomedicine ultimately relies on the transition from *in vitro* NP–PC characterization into *in vivo* systems.

**Fig. 4:**
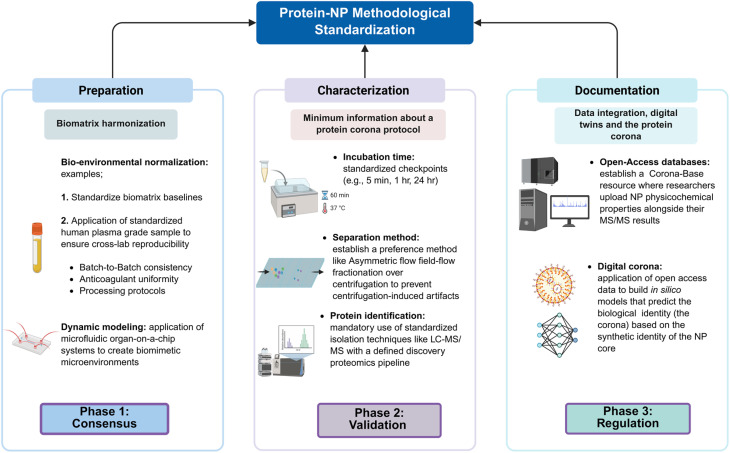
Scheme of proposed framework for NP-protein corona methodological standardization. The top panel shows three pillars: preparation, characterization, and documentation of the protein corona and NP. The bottom panel (phases) shows the implementation roadmap for the three approaches. Phase 1 (Consensus): establishing a reference plasma and minimum working protocol through international consortiums, Phase 2 (Validation): multi-center characterization of the NP-PC across a defined minimum of global labs to ensure reproducibility and community consensus, and Phase 3 (Regulation): integrating NP-PC characterization into chemistry, manufacturing, and control platforms for regulatory approval.

Furthermore, to the best of our knowledge there is almost no community-led initiatives or guidelines on how the protein corona should be documented for approval. This underscores that some regulatory and quality control roadmaps should be implemented in the NP-PC methodological standardization to predict and reproduce protein corona reports (Fig. 4). In one notable example, the Morteza Mahmoudi group employed standardized protocols and unified sample preparation workflows. Their findings demonstrated a significant enhancement in the consistency of protein corona data. Specifically, the use of standardized workflows increased protein identification overlap from 11% to 40% across multiple facilities using identical instrumentation and search parameters.^191^

In the context of neurodegenerative research, such as Alzheimer's or Parkinson's disease, the PC presents unique hurdles that current methodologies struggle to address: BBB translocation, protein misfolding and associated toxicity, and compromised barrier dynamics. The first difficulty lies in capturing the true composition of the PC inside the brain. This is because the PC composition evolves dynamically and drastically upon crossing the BBB. Consequently, methods that only characterize the corona in the bloodstream fail to account for the brain-specific corona acquired beyond the barrier, which ultimately dictates how NPs interact with neurons.^120,192^ Indeed, techniques such as pre-incubation in serum overlook the dynamic, continuous exchange of proteins that occurs over time. Furthermore, since adsorption onto a nanoparticle surface can cause proteins to denature or unfold, the effects of protein misfolding and toxicity must be considered in methodological standardization, specifically in neurodegenerative contexts. Most importantly, in diseased states, conditions such as neuroinflammation promote BBB permeability, causing proteins like albumin to leak across the barrier into the brain. However, standard experimental designs typically utilize a healthy BBB, failing to capture how a diseased environment alters corona formation and nanoparticle biodistribution.^193^ Furthermore, it is imperative to note that most NP characterization reports fail to account for the effect of PC on fibrillogenesis of amyloidogenic proteins, such as amyloid-β, tau, and α-synuclein.

Therefore, bridging the gap between laboratory research and clinical success requires a more holistic approach to NP characterization. There is a need for dynamic methods capable of tracking PC evolution during systemic transport. *In-situ* characterization must account for how pathological states, like a diseased brain environment alter the PC coating and composition. Furthermore, the influence of the NP surface on protein structural changes and the potential for misfolding into toxic aggregates must be addressed.

### 3.4. Possible strategies to address challenges of protein corona formation

The effect of protein corona on NPs application cycle from the injection process to the degradation is the major hurdle. Considering the general steps of NP-protein corona characterization (Fig. 3), any limitation or misinformation in either of the steps can be a source of errors.

In line with this, collection and storage of biological fluid is a pivotal step in the nanomedicine development in which misinformation or inadequate information can create pronounced error in the downstream processes that could potentially result in inappropriate NP protein corona data analysis. Concomitantly, the protein source, pre-processing conditions including lysis conditions, incubation and serum/plasma treatment and media used for washing absolutely affects the PC composition and cellular uptake, and therefore are fundamental steps towards the effective *in vitro* protein corona analysis.^126^ For instance, factors including choice of anticoagulant,^128^ long term storage of plasma samples at −80 °C^194^ and temperature^90^ heavily affect the protein corona composition.

Asides the common sources of poor corona data analysis in the biosystem, another challenge relies on the techniques and methodologies utilized for characterization of physicochemical properties, colloidal stability of corona-coated NPs and PC composition, all of which are key to accurately predict the biological fate of NPs.^195,196^ Thus, to precisely characterize protein corona the fundamental understanding of the sources of information and the choice of suitable techniques is critical.

#### 3.4.1. Protein-repellent coating and pre-coating strategies

In this approach, molecules that can act as protein repellent to prevent or reduce the shielding effect of corona protein can be employed to achieve improved colloidal stability, prolonged circulation time, better targeting capability and minimal immunogenicity.

One common strategy is nanoparticle surface functionalization using antifouling polymers such as polyethylene glycol (PEG) conjugation (PEGylation).^197–199^ Because of its biocompatibility, and sustained release properties, PEG is one of the most widely used molecule in the NP surface modification strategies to develop controlled and targeted drug delivery systems.^200,201^ Based on density, size, length and heterogeneity of the polymeric coating,^202^ NPs functionalized with PEG molecules significantly reduced macrophage cellular uptake, performed better targeting efficiency and notable reduction of bound proteins.^203–205^ However, the non-degradable nature of PEG causes multiple side effects and repeated intravenous administration of NPs such as gold nanoparticles leads to PEG antibodies (anti-PEG Abs) generation and, thereby, to rapid NP clearance.^206,207^

Molecules that create a zwitterionic surface on nanoparticles are also used as protein-repellent coating.^18,208^ For instance, nanostructures which have a protective shell such as surface enhanced Raman scattering tags become a promising tool to reduce PC formation. Experts in the field demonstrated their applicability by synthesizing small cyclic arginine-glycine-aspartic acid-phenylalanine-cysteine (RGDFC) peptides functionalized on the surface of spherical gold nanoparticles, that showed resistance to corona formation.^209^

Another employed approach to manipulate the protein corona composition is pre-coating the nanoparticle surface so as to recruit the proteins that can increase targeting efficiency of NP.^210,211^ One notable example is the use of affibody (RA) scaffold and glutaraldehyde (GA) to load trastuzumab (TZ) on to magnetosome that showed to recognize and target cancer cells even in the presence of protein corona.^212^ In a simulated *in vivo* milieu, Ma *et al.* demonstrated that the RA promotes and orients the arrangement of targeting ligands and reduces the shielding effect of corona proteins that improved the targeting capability and drug delivery of NP.^212^ Similarly, Chitosan (Ch)-based nanoparticles, including Ch and carboxymethyl dextran or thiolated dextran polyelectrolyte complexes, accumulate low amounts of proteins corona that improves uptake of nanoparticles, and exhibit low liver uptake and notable heart blood pool accumulation.^213^

Other proteins such opsonin, which help immune cells to engulf particles, are also hypothesized to control the phagocytosis process.^214^ On the contrary, pre-coating NPs with specific PC proteins that lack opsonins was showed to reduce association of NPs with leukocytes.^215,216^

Since different surface coatings change the PC size and composition, characterization of NPs before and after corona formation and detailed studies on the PC are of utmost importance to determine the most suitable NP surface modification for biomedical use.

#### 3.4.2. Conjugation to protein corona strategies

Several proteins are preferentially bound to either hydrophobic (*e.g.*, vitronectin) or hydrophilic NP (*e.g.*, haptoglobin), providing eligibility of the NP for potential therapeutic or diagnostic use.^72^ From interaction study with l-methionine capped silver nanoparticles (AgMet), bovine serum albumin (BSA) exemplified as a potential external stabilizer agent.^217^ Though, BSA can cause “BSA corona-caused aggregation” as noticed in solid lipid nanoparticles (SLNs) with increasing particle size of 120 to 480 nm and different pH,^43^ affecting secondary structure of BSA and cellular uptake of SLNs. In other corona modified nano-delivery systems (NDS) study, conjugation of BSA on a chitosan core was used for sustained and effective delivery of carvacrol under the influence of various simulated gastrointestinal conditions. Accordingly, the BSA corona provided extra stability to NDS by maintaining positive zeta potential, ensuring delayed release and limited degradation in the gastric conditions, and reduced the mucoadhesion of NDS at gastric pH.^218^

Another related emerging strategy is pre-adsorption of antibodies by means of physical approach (physisorption) to attach target moieties to nanoparticle surface.^210^

#### 3.4.3. Protein modification strategies

Modification of proteins can be used as alternative strategy to reduce the re-absorption of protein corona and minimize the adverse effect of protein corona on targetability. One such notable example of this is the human serum albumin (HSA), one of the most abundant protein in blood plasma, and important component in the protein corona.^219,220^ With the potential of preventing nanoparticle aggregation,^221^ its availability and surface modification makes HSA suitable for specific applications including cancer targeted drug delivery.^222,223^ For example, Yang *et al.* were able to demonstrate remarkable reduction in reabsorption of protein corona from blood serum using fluorescein-isothiocyanate and folic acid-modified human serum albumin shell on hybrid nanomaterials with bismuth sulfide (Bi(2)S(3)) nanorods.^224^ Interestingly, it is also mentioned that conjugating ligands to an equilibrated nanoparticle-corona can help to enhance the ligand-targeting function, especially when compared to direct ligand conjugation to the naked NP surface in serum.^225^

### 3.5. Protein corona cellular uptake, trafficking, and immune response

In the rapid development and progress of nanotechnology and nanomedicine, numerous efforts has been made by scientists to investigate the application of NPs for delivery of large-cargo molecules through the cell membrane for diagnostic and therapeutic purposes.^6,226–228^ It is also widely believed that the entry of NPs into the cell interior is a critical step to improve intracellular delivery of pharmaceuticals.^229–231^

However, as extensive research documents, the blood circulation, accumulation and penetration of NPs at targeting sites could be influenced by the formation of protein corona, in either negative, or positive ways. This ultimately defines the cellular uptake, biodistribution, pharmacokinetics, cell interaction, and toxicity of nanoparticles. Furthermore, the cellular uptake in different diseases targeting delivery and the interactions between NPs and receptors on immune cells for immunotherapy could be also influenced.^232–234^

**Fig. 5:**
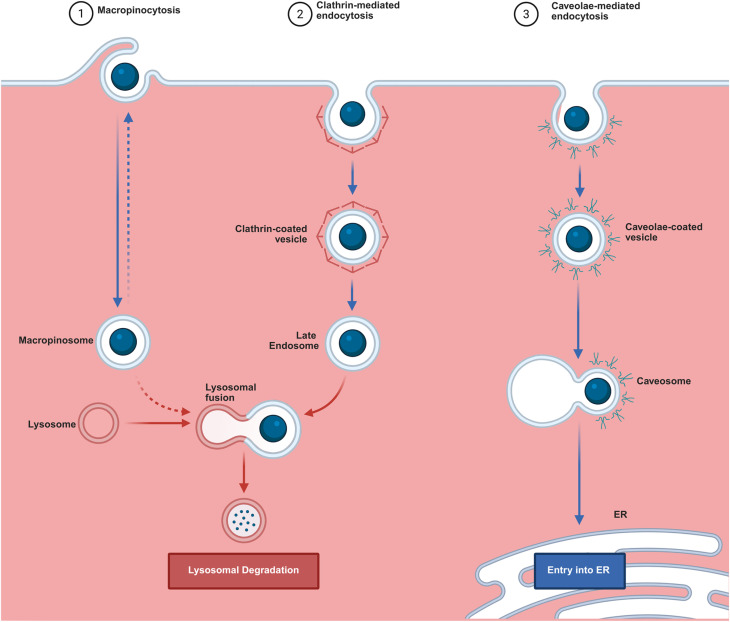
General mechanistic pathways and intracellular sorting of NPs. Larger NPs and bulk extracellular fluid are internalized *via* macropinocytosis, whereas small-to-mid-sized NPs predominantly enter through specialized receptor-mediated pathways, such as clathrin- and caveolae-mediated endocytosis. (Left) Macropinocytosis and (Center) Clathrin-mediated endocytosis involve the uptake of particles into vesicles that mature into the endolysosomal system. The transition from blue to red arrows during lysosomal fusion indicates the acidification of the compartment that ultimately leads to the lysosomal degradation of the NPs. On the other hand, (Right) Caveolae-mediated endocytosis provides a non-degradative alternative. As indicated by the solid blue arrows, nanocarriers in this pathway mostly bypasses the acidic lysosome, thereby trafficking *via* the caveosome to the Endoplasmic Reticulum (ER). Dashed blue lines indicate secondary trafficking events, such as surface recycling or endosomal escape.

The cell membrane is selectively permeable allowing materials to enter into the cell, either *via* active transport or passive diffusion.^235^ As such, the process of NP uptake relies on these two mechanisms.^236^ In general, cellular uptake of nanomaterials can be categorized in to three endocytic pathways namely receptor-mediated endocytosis, receptor-independent pathway and micropinocytosis.^235,237^ Clathrin-mediated endocytosis is a major pathway for receptor-mediated endocytosis. Receptor-independent pathway can be further classified as caveolin-mediated endocytosis, clathrin-independent endocytosis, clathrin- and caveolin-independent endocytosis (Fig. 5).^238,239^

It is well understood that the cellular uptake of NPs is not only determined by the nature of cell membrane but also the NPs properties itself.^240,241^ For this reason, each type of nanoparticles are expected to be internalized *via* different endocytosis pathways based on their physico-chemical features,^242,243^ on the biological microenvironment,^244,245^ and on the adsorbed protein corona.^232,242^ Interestingly, a work done by Ding and his colleagues clearly demonstrated the key role of size, shape, and protein corona in cellular uptake mechanisms of gold nanoparticles.^246^ According to this study, NPs with size of 15 nm, 45 nm, and the rod-shaped NPs were internalized into cells *via* a receptor-mediated endocytosis pathway. Moreover, while the star-shaped NPs adopted both clathrin-mediated and caveolin-mediated endocytosis pathways, the 80 nm sized NPs were internalized into the cells by macropinocytosis pathway.^246^ Other group of researchers also demonstrated that the protein corona on 40 nm polyethylene glycol coated AuNPs altered the internalization mechanisms mediated by clathrin.^247^ In line with this, recent study reported 20 nm sized AuNPs inhibition of macropinocytosis endocytic pathways.^248^ From time point evaluation of protein corona composition on 15 nm citrate stabilized gold NPs, Wang *et al.* were able also to demonstrate clathrin-mediated uptake as main endocytosis pathway of AuNPs.^110^ Moreover, a systematic study on the internalization of AuNPs of various size, shape, and surface coating, reported reduced cellular uptake in the presence of serum. While regarding the shape of particle, rod shaped were better internalized followed by cubic, spherical, prism-like AuNPs.^249^ In general, the most common route of NPs uptake in non-specialized mammalian cells is the clathrin-mediated endocytosis.^238^ Specific interactions with negatively charged NPs may also result in caveolin-mediated or clathrin-mediated endocytosis.^250^ In non-specific cell membrane interactions, smaller NPs have a greater chance of being engulfed by macrophages compared to larger NPs, which will be taken by pinocytosis.^235^

In summary, given the dynamic protein composition of protein corona, the mechanistic evaluation of cellular uptake of NPs is quite complex. In one hand, endocytosis processes are intrinsically temperature-dependent,^93,94^ suggesting that working in different incubation temperature would be of great importance to understand evolution of protein corona. On the other hand, physico-chemical properties of NPs will influence protein corona adsorption, thereby dictating the cellular uptake and intracellular trafficking.^251,252^

For these reasons, cellular uptake of NPs it is continuously being aided by the design of nanocarriers with desired physico-chemical properties.^6,253^ For example, NPs superficial modification aids to utilize particular interactions between NP superficial ligands and the existing receptors on the cells, so that facilitates active targeting and cellular uptake.^254–256^ It was also demonstrated that rod-shaped AuNPs showed more elevated membrane adhesion efficacy compared to spherical AuNPs.^257^ Furthermore, increased cellular internalization was observed proportional to positively charged NPs,^258,259^ which could reverse the decreased uptake of negatively charged NPs.^260^ In a similar approach, cationic and anionic parts of NPs were manipulated to bind to the targeted receptor and the protein receptor, respectively.^259^ Therefore, to achieve a desirable cellular uptake efficiency, considering these elements in every step is indeed *a* point to be highlighted.^261^

It is well understood that the protein corona formation promotes the interaction with cells and thereby influences the cellular uptake of nanoparticles, in either positive or negative ways. Indeed, the nano-bio interface can dictate nanoparticles to modulate cellular structures, intervene cell–cell communication and many other biological processes. Thus, protein corona caused shielding of targeting groups and displacement of initial protein on the nano-surface could cause the loss of active targeting and could also lead to disruption of major cellular processes from disruption of key cellular metabolism pathways to cell death.^262,263^ The protein corona surrounding NPs in serum contains proteins such as complement, immunoglobulins, and apolipoproteins that can trigger the immune system.^264^ Complement proteins deposited on NP surfaces could potentially make them more susceptible to removal by immune cells, which could possibly lead to some negative effects.^265^ When PC-mediated immune system captures nanoparticles, it can have a negative impact on their systemic half-life, delivery capability, and overall safety profile thereby causing immune perturbation.^52^

Overall, understanding, predicting, and manipulating the nanoparticle protein corona and its interaction with immune system-related cells is of the utmost importance in development of drug delivery nanoplatforms.^11,266^ As a matter of fact, precise investigation of interactions of NPs with cells could be extremely challenging due to the existence of numerous cell types and multitudes of key environmental factors. As we highlighted in the previous sections, manipulating the NPs features can also be applied to alter NPs tropism for immune cells. However, the modifications of NPs are complicated and costly and make the NPs even harder for industrialization.

## 4 Protein corona amyloid protein interaction and neurodegenerative diseases

It is widely recognized that the main pathophysiologic pathway for the onset and progression of Alzheimer's disease is the aggregation and fibrillation of Aβ protein.^267,268^ Over the past few decades, several experiments have been conducted to identify an effective approach to inhibit Aβ aggregation and fibrillation, as it could potentially halt or even prevent the progression of the disease.^269^ For that purpose, NPs have been gaining recognition as a promising approach to cease the progress of the disease by regulating the fibrillation kinetic process.^270,271^ In fact, studies have proven that NPs can modulate protein aggregation and fibril formation in the context of amyloid diseases.^14,272,273^ The reason why NPs would have an advantage over anti-amyloidogenic small molecules is their controllable size and surface chemistry, especially when compared to anti-amyloidogenic small molecules (having single amyloid binding sites and weak binding capability).^274,275^ Specifically, the design of NPs allows them to provide multiple binding sites, a strong binding event, and delivery across physiological barriers, making them indeed a promising option.^269,276^

**Fig. 6:**
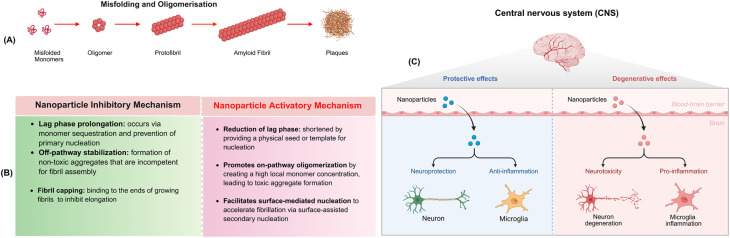
Nanoparticle-mediated effects in the central nervous system. (A) Amyloid aggregation pathways. (B) Possible effects of NPs on each step of amyloid aggregation pathways that could lead to either inhibitor or activator effects. (C) The dual nature of NPs interactions within the brain, categorized into protective effects (left) and degenerative effects (right). Left Side: NPs can provide neuroprotection and anti-inflammation mostly through microglial modulation, neuroregeneration and other mechanisms. Conversely, NPs exposure can also trigger pro-inflammatory microglia and subsequent neurodegeneration. As illustrated in the right side, neurotoxicity and pro-inflammation can be driven by chronic neuroinflammation (such as over-activation of microglia), protein aggregation (for instance, the presence of specific metallic NPs like ZnO or Ag) and can accelerate the misfolding and aggregation of pathological proteins such as Amyloid-beta or Tau), and other pathological mechanisms.

The NP surface acts as a heterogeneous catalyst that will induce a conformational switch of the amyloidogenic proteins. As such, NPs may act as pro-fibrillatory, where the surface concentrates proteins, increasing the local density and lowering the energy barrier for nucleation. On the contrary, the NP may also act as anti-fibrillatory by sequestering the monomers, keeping them trapped in a non-amyloidogenic state or sterically hindering the addition of new monomers to a growing chain (Fig. 6).^92^

The fibrillation of amyloid proteins follows a three-stage kinetic model characterized by nucleation, elongation, and saturation phases; during this process, monomers undergo primary nucleation to form intermediate oligomers and protofibrils before finally assembling into mature amyloid fibrils (Fig. 6).^277^ In primary nucleation, the spontaneous formation of the first aggregate seeds from monomeric proteins. NPs often act as a heterogeneous surface that lowers the energy barrier for this step. On the other hand, during the elongation phase, NPs act as competitive inhibitors; an NP with a high-affinity corona can sequester monomers, reducing the concentration available for fibril elongation.^111^ Conversely, during secondary nucleation, new nuclei form on the surface of existing fibrils rather than in the bulk solution. At this stage, the NP can coat the existing fibrils *via* steric hindrance, thereby blocking the sites needed for secondary nucleation.^278^

The interaction between NPs and amyloidogenic proteins is a dynamic interplay of surface chemistry and molecular kinetics. As such, selective adsorption of nanoparticles, and their biological identity, and the conformational modification of the amyloidogenic protein determines the molecular interplay between the protein corona and pathologic proteins, including Aβ, tau, and α-synuclein.^279^ For instance, the amphiphilic properties of the corona, driven by its lipid and protein composition like apolipoproteins, allow it to act as a modulator of the local peptide concentration and conformational changes of Aβ.^14^

The Aβ fibril growth is not a continuous addition of monomers. Instead, it consists of intermittent periods of elongation and pausing that follows a ‘stop-and-go’ kinetics.^280^ A study by Yagi-Utsumi *et al.* demonstrates a kinetic model in which a protein first docks or associates with the fibril end or a surface such as a NP before locking into the final β-sheet conformation,^280^ highlighting the pausing phase is a critical kinetic bottleneck where inhibitors like nanoparticles or antibodies can most effectively intervene. Within the same scenario, the N-terminal amphipathic motifs (KA/TKE/QGV) of α-synuclein initiate an enthalpically driven adsorption onto the NP interface while the subsequent bulk-protein adsorption onto the hard-corona triggers a conformational switch.^281^ For instance, while elevated concentrations of α-synuclein exhibit stronger affinity for the negative surface potential of a ZNO NPs,^281^ these interfacial interactions induce a shift in the aggregation landscape, kinetically trapping the protein into off-pathway, non-toxic species that lack the inherent pathogenicity of mature fibrils.^281^ Similarly, anionic proteins in the hard corona undergo induced fit to bind the microtubule-binding repeats of Tau protein. Mainly the electrostatic forces (anionic/cationic interactions) regulate the interaction of Tau nanocondensate with hard corona of anionic proteins, triggering a cascade of structural and functional changes that will result in either failure within the neuron or promote Tau protein in a functional physiological state.^282^

A recent review article on kinetics of amyloid oligomer formation demonstrates how chemical kinetics is used to determine whether a modulator (like NPs) affects primary nucleation, secondary nucleation, or elongation.^283^ These findings suggest that while nanoparticles accelerate the ‘go’ phase of the ‘stop-and-go’ cycle by offering stable docking sites, the protein corona can counteract this effect. By acting as a competitive inhibitor, the corona traps the amyloidogenic protein in a paused state, effectively blocking the transition from docking to final fibril incorporation.

Notable findings from studies related to NPs and amyloid fibrillogenesis indicate that NPs can have varied effects on amyloid fibrillogenesis, as promoters, or even delay/inhibit the process, and these effects are influenced by various factors such as sequence of the polypeptide, physicochemical conditions present, as well as the physio-chemical properties of the NP.^284–286^ One hypothesis regarding NPs role in accelerating the rate of fibril formation is that NPs provide a seeding surface for the adsorption of peptide monomers, enabling nucleation to oligomers and fibril formation.^285^ Likewise, adsorption of proteins onto the NP surface could deplete their concentration in solution, which may inhibit aggregation as well. Overall, there still remains a lot to learn regarding the characteristics of NPs and how they interact with various proteins to gain a comprehensive understanding of their influence on amyloid aggregation.

### 4.1. Anti-fibrillation effect of nanoparticles

A crucial point to discuss is the impact of the physicochemical properties of a nanoparticle, such as its size,^287^ charge,^287^ and shape/morphology,^288^ on its ability to inhibit or promote amyloid fibril formation. A study done using gold NPs coated with various substances such as citrate, CTAB (*cetyltrimethylammonium bromide*), PAA [poly (acrylic acid)] or PAH [*polyelectrolytes poly (allylamine)hydrochloride*], showed that size and surface chemistry of NPs determine their inhibition ability, as well as surface charge determines the morphological features of the aggregates.^289^ Similarly, silver NPs caused rapid dissolution of fibrils, and in comparative experiment performed, the *poly(vinyl) pyrrolidone (PVP)-*stabilized negatively charged *triangular silver nanoplates (AgTNPs)* were found to be more effective than the PVP-stabilized silver nanospheres, and dissolved the fibrils at 1 hour and 70 hours respectively.^290^ Another experimental study focused on the effect of different nanoparticle morphologies on fibrillation/antifibrillation: Giannousi *et al.* synthesized two types of ZnO nanoparticles (*ZnO nanoflowers and polyol-coated ZnO NPs*), revealing that both nanomaterials impacted the amyloid formation mechanism and disaggregation, with ZnO nanoflowers, which have sharp edges, showing the greatest amyloid degradation rate.^291^ Moreover, surface modification of NPs could increase the efficacy of the anti-fibrillation effect. As such, selenium NPs conjugated with targeting peptides were found to be more effective for inhibiting the aggregation process of Aβ1–40, *via* a synergistic effect that blocks the active site of fibril formation.^292^

So far it is well studied that NPs have the ability to inhibit the formation of fibrils by preventing the assembly of monomers and oligomers, and this is achieved through altering hydrophobic interactions or with specific ligands; they could also bring conformational changes in monomeric species too (Fig. 6).^269,293^ Especially gold nanoparticles (*AuNP*) have gained popularity as efficient inhibitors of fibrillation owing to their unique features such as inert behavior, and tunable structural and chemical properties.^293,294^ Recently, a fascinating technique is being developed to prevent amyloid fibrillation by designing a nanoparticle cluster, which uses a point-to-point strategy to expose more binding sites in different types *via* multivalent binding: once this cluster reaches the AD nidus, it decomposes into ultra-small nanoparticles and binds with the Aβ sequence preventing aggregation.^295^

NIR (near infrared) light-responsive NPs have an added advantage as they can efficiently penetrate the blood–brain barrier (BBB), and subsequently inhibit amyloid-β1–42 (Aβ1–42) fibrillation and disaggregate fibrils, by exhibit extremely strong binding affinity for the Aβ1–42 protein.^269^ It has also been stated that the strong bond between nanoparticle and amyloid competitively reduces amyloid–amyloid interactions thereby disintegrating amyloid fibrils, eventually forming (NP-&-Aβ) complex, thereby further normalizing microglial immunologic dysfunction for Aβ removal.^295^ Moreover, Sutherland *et al.* also proposed the mechanism of inhibition of insulin amyloid fibrils, stating that the biopolymer-coated AuNPs strongly interact with the insulin monomers and inhibit the oligomer formation as well as elongation of the protofibrils.^296^ Furthermore, cytotoxicity experiments showed that AuNP-insulin amyloid fibrils are less toxic compared to insulin amyloid fibrils alone. NPs could also destroy preformed proto-fibrils, as shown by the hydroxylated single-walled carbon nanotube (*SWCNT-OH*) that showed not only excellent anti-amyloid properties, but, also the attachment of β2-microglobulin (β2m21–31) on its surfaces adopting unstructured formations, which impede the fibrillation process.^297^ By employing a mechanistic model, Liu *et al.* proposed that hydrophobic binding and electrostatic repulsion are mainly responsible for the interaction between Aβ1–42 and NPs, then, the ‘*Aβ1-42-copolymeric NPs interaction*’ leads to the stretching of Aβ1–42 molecules avoiding the formation of fibrillogenic β-sheet structures.^298^ Similarly zinc oxide (ZnO) NPs inhibited fibril formation by decreasing cross-β sheet amount, and preventing an increase in surface hydrophobicity.^15^

Another side of the nanoparticle–amyloid interaction is how and at what stage of the fibrillation NPs act on it, as the mechanisms of action can vary and are dependent on the specific target.^299^ For instance, polymeric nanoparticles affect the nucleation step which is the initial stage of fibrillation process.^300^ The starch-capped ZnO (*ZnONPST*) NP prolonged the nucleation phase and shortened the elongation phase of amyloid growth.^301,302^ Counterintuitively, redirecting the amyloid fibril formation toward non-toxic pathways is also another therapeutic strategy. NPs might prevent the formation of smaller, more toxic oligomeric species by accelerating the formation of large stable fibrils, and thereby positively affect the (initial) progress of amyloid-related diseases.

Surface modifications of NPs could also help to enhance the efficacy of anti-fibrillation process. The inhibitory activities appear to stem from the favorable interactions between modified NPs and early pre-amyloid species.^271,303^ For example, in the case of Citric acid surface-modified magnetite nanoparticles *(COAT-MNPs),* the interaction potentially reduces the formation of nuclei and oligomers that are necessary for amyloid fibrillation^304^ while curcumin-conjugated silver NPs inhibit the amyloid fibrillation of lysozyme by restricting the formation of larger fibrils.^305^ The presence of certain amino acids on the surface of AuNPs might have the potential to control the aggregation of proteins.^271^ As such, strategically synthesized stable gold (*AuNPs (Tyr), AuNPs (Trp)*) and silver (*AgNPs (Tyr)*) NPs surface functionalized with either tyrosine or tryptophan residues showed inhibition of both spontaneous and seed-induced aggregation of insulin, and triggered the disassembly of insulin amyloid fibrils.^303^

### 4.2. Fibrillation effect of nanoparticles

It's interesting to note that while the impact of NPs on anti-fibrillation has been discussed, there seems to be a contradiction when it comes to the effects of NPs on amyloid fibril formation. Studies have shown varying results, and more exploration is needed in this area. What we do know is that NPs may either promote, delay, or inhibit amyloid fibrillogenesis, depending on their properties. This is worth considering when administering nanomaterials for brain delivery, as they may promote aggregation and lead to amyloid fibrils.

As such, poly (*propylene imine) (PPI*) dendrimer, lacking a positive charge,^306^ polyethylene terephthalate (PET)^307^ nanoparticles promoted amyloid fibril formation. Besides, as shown by molecular dynamics simulation, TiO_2_ NPs can adsorb on the surface of the Aβ_42_ peptide and accelerate its oligomerization, by stabilizing the binding site with hydrophobic interactions.^308^ This is also supported by other findings, that demonstrated hydrophilic TiO2 may have an effect on the formation and aggregation of Aβ42 fibrils by shortening nucleation stage.^112,309^ Furtherly, Surface-Enhanced Raman Spectroscopy (*SERS*) and Tip-Enhanced Raman Spectroscopy (*TERS*) experimental study showed that TiO_2_-NPs and ZnO-NPs exposure to amyloid protein caused morphological changes and stimulated aggregation and fibrillation kinetics of β-amyloid fragment 1–40 (βA) and α-synuclein protein after incubation at 37 °C.^310^ In addition, it has been observed that the interaction between human islet amyloid peptide (*hIAPP*) and MoS2 nanosheet results in a specific nano-bio interface phenomenon, which is enabled by the MoS2 nanosheet's ability to attract hIAPP monomer, dimer, and oligomer on its surface through van der Waals forces.^311^

Interestingly, amyloid formed in the presence of ZnONP actually exhibits significantly reduced cellular toxicity when compared to pure amyloid, that was found to have profound cellular toxicity in both mouse carcinoma N2a and normal cells, such as human keratinocytes HaCaT cells.^301^

For the factors mentioned above and other reasons, so far, the studies done on the effect of NPs on amyloid fibrillation had shown varying results (*promotory/inhibitory*), thereby when studying the effect of NPs on amyloid fibrillation, the physico-chemical characteristics of the NPs need to be considered. Sideways, it is important to note that nanomaterials can have serious health implications if they enter the bloodstream and make their way to other organs, such as the brain. The reason for this concern is that nanomaterials have a large surface area and strong interaction energy, which allows them to absorb proteins in their surroundings. Therefore, whenever administering NPs in an experiment, making precaution is always cardinal.

**Fig. 7:**
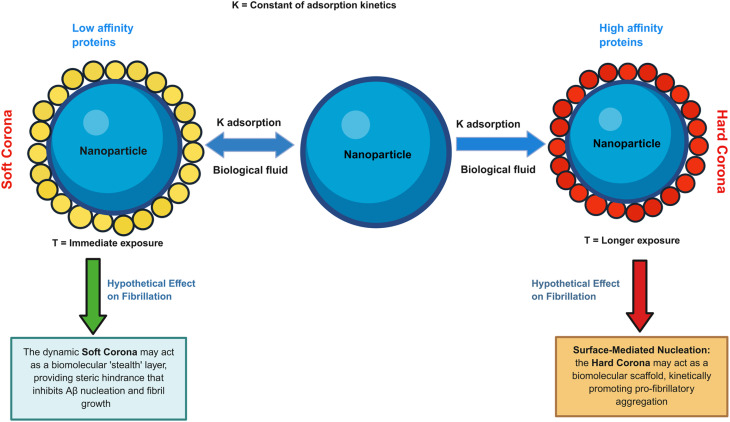
Schematic illustration of kinetic aspects of protein corona formation. It depicts the time-dependent evolution of the soft and hard protein corona on NPs that consequently could hypothetically lead to either anti-fibrillatory or pro-fibrillatory aggregation effects on fibrillation process of amyloidal proteins, respectively. Figure reproduced with minor modification from ref. 312.

As discussed earlier, the complex interplay between the NP surface, the protein corona, and the bulk biological fluid determines the corona formation. When amyloid proteins (like β-amyloid or α-synuclein) encounter a NP, they bind to the surface based on hydrophobicity, charge, and curvature. This will eventually lead to the formation of either the hard corona (*i.e.*, proteins bound with high affinity, often undergoing significant conformational changes) or the soft corona (*i.e.*, loosely associated proteins that exchange rapidly with the environment). Consequently, such an NP–PC complex may play either an anti-fibrillatory or a pro-fibrillatory role in the fibrillation process of amyloid proteins (Fig. 7).^92^

### 4.3. Overview of amyloid protein–protein corona interaction, and its effect on oligomerization and fibrillation

While numerous studies have focused on the impact of nanoparticles (NPs) on oligomerization and fibrillation processes, most of these studies have overlooked the influence of the biomolecular corona on the fibrillation process. Given that the protein corona plays a crucial role in determining the biological fate of NPs, it's essential to investigate its molecular dynamics thoroughly.

When NPs are administered *in vivo*, the delivery of NPs and their interaction with Aβ protein is not pristine anymore, rather protein coated, which can affect the NPs to either promote or inhibit fibrillation processes.^313^ For instance, PEGylated polystyrene NPs and transferrin-modified NPs lose their active targeting characteristics towards bEnd.3 and C6 cells when incubated with CSF *in vivo* and *in vitro*.^314^ Therefore, an emerging field nowadays is to try to understand and harness the NPs-PCs-amyloid proteins interaction as a whole, rather than solely the NPs-amyloid proteins interaction. Making a study in this whole-round approach will not only enable us to understand the biological effects of nanomaterials, but also can help us get a deep insight into the evolution of amyloid diseases.

The assembly of fibrils proceeds *via* primary nucleation and elongation, incorporating secondary mechanisms like secondary nucleation and fragmentation.^283^ Thus, understanding the inhibitory role of the protein corona requires an analysis of the competitive adsorption kinetics occurring at the nanoparticle interface, which can effectively sequester monomers away from the fibril's elongation front.

In this context, the protein corona actively interferes the ‘stop-and-go’ mechanism of fibrillation.

**Fig. 8:**
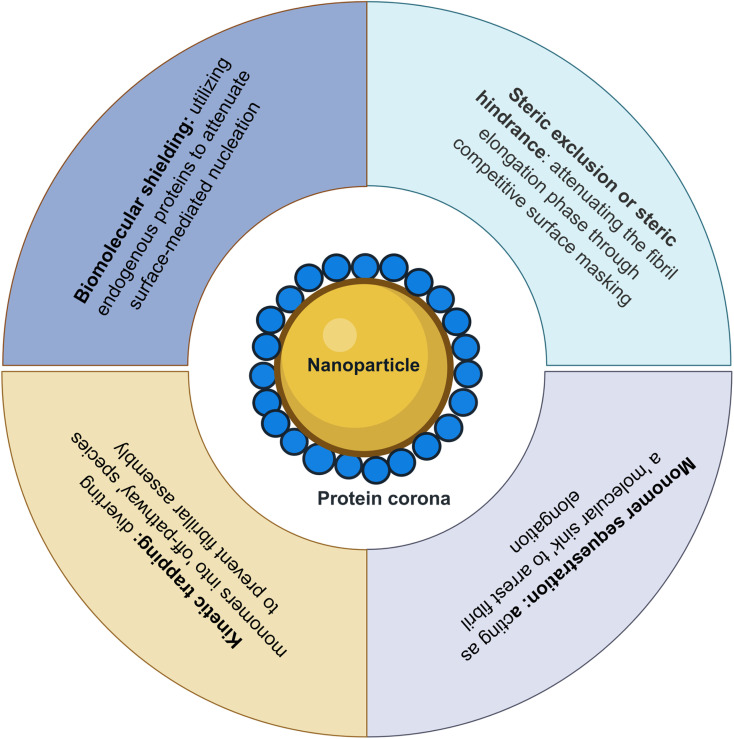
PC competition against amyloidogenic proteins for the NP surface.

Here we highlight some possible mechanistic models on the mechanism of a protein corona competition against amyloidogenic proteins for the NP surface, and resulting possible effects on the fibrillation (Fig. 8). The first mechanism is steric hindrance (or steric exclusion), which enables a protein corona mask reactive sites on the nanoparticle surface. For instance, when non-amyloidogenic proteins like albumin cover the NP surface, they physically block amyloid monomers from reaching the surface where they would otherwise nucleate.^315^ A second possible mechanism is that the protein corona acts as a monomer sink by sequestering the building blocks of the fibril. By pulling monomers into the corona, the NP effectively starves the growing fibril of the precursors required for elongation, thereby slowing the rate of fibrillation.^14^ A third possibility is that the protein corona induces kinetic trapping, creating off-pathway oligomers. When amyloidogenic proteins enter the corona, the NP surface may stabilize a conformation distinct from the native or β-sheet folds. These structurally incompatible oligomers cannot join the fibril, resulting in an accumulation of off-pathway species instead of fibril growth.^14^ Last but not least, specific proteins may act as molecular chaperones or competitive guards against pathological proteins, leading to the inhibition of key steps within the amyloid aggregation pathways.^14,316^

Based on the above hypothesized mechanistic insights, monitoring the fibrillation process of amyloidal proteins in the presence of corona-coated NPs, rather than NPs only, could help to achieve a more reliable and predictable outcome.

Likewise, the targeting specificity and cellular uptake of NPs could also be shielded by protein corona from serum albumin or CSF,^317^ and so far researchers have identified 58 proteins that are exclusive to Aβ1–42 samples and 31 proteins exclusive to Aβ1–40 samples in human blood plasma, significant in the AD pathology, with an impact on complement activation, inflammation, and protein metabolic pathways.^109^ Interestingly, when amyloid fibrils are *in vivo*, they also form a PC, which alters their biological identity according to an experimental study done on the characterization of the PC of human islet amyloid polypeptide (IAPP) fibrils in FBS (fetal bovine serum) which revealed a clear evidence for the formation of a significant protein corona.^314^ This will take us to a new perspective according to Pilkington *et al.* statement,^318^ which affirms that the formation of amyloid coronae can help to reduce the toxicity of the fibril, which could be a promising new mechanism for mitigating IAPP toxicity *in vivo*.

Generally, according to several studies, protein corona creates a shell over different nanomaterials, resulting in reduced levels of amyloid β-fibril formation, compared to pristine nanomaterials.^111,319^ In complement to these findings, the binding of islet amyloid polypeptide (IAPP) with two homologous proteins, cationic lysozyme (Lys) and anionic alpha-lactalbumin (aLac), also reinforced the statement that protein “corona” plays a crucial role in conferring the biological impact of amyloidogenic peptides.^320^

Simultaneously, the inhibitory strength of NPs is determined by the amount of proteins that are adsorbed onto their surface which ultimately also shows the significance of the surface properties of NPs in determining their effect on altering fibrillation process. Furthermore, an experimental study done on the effect of PC coated NPs on the fibrillation of *α-Synuclein* showed that the primary nucleation becomes slower, as PC reduces the accelerating effect of NPs, which eventually results in the formation of short fibrils and long fibrils in the absence and presence of PC respectively.^111^ Similarly, protein corona formed a shell at the surface of gold NPs, regardless of their size and shape, reducing the access of Aβ to the gold inhibitory surface and, therefore, affecting the rate of Aβ fibril formation: specifically, the anti-fibrillation potencies of various corona-coated gold NPs were strongly dependent on the protein source and their concentrations.^321^

However, the dynamicity of the PC-amyloid protein remains complex, as opposite results were obtained from a study demonstrating that biomolecular corona can dictate either an inhibitory or acceleratory effect of NPs on fibrillation processes. Their finding stated that biomolecular-corona-coated gold NPs has less inhibitory effect on Aβ_1–42_ fibrillation kinetics, while good inhibitory effect on Aβ_25–35_ peptide.^322^ Interesting mechanistic convergence between viral and amyloid pathologies has also been elucidated, like respiratory syncytial virus (*RSV*) and herpes simplex virus type 1 (*HSV-1*) accumulate a rich and distinctive protein corona. In *in vitro* and animal model studies, it has been shown that amyloidogenic peptides bind in the corona and catalyze amyloid formation *via* surface-assisted heterogeneous nucleation.^323^

Overall, NP surface acts as a competitive interface where plasma proteins and amyloidogenic monomers undergo dynamic exchange for adsorption sites. Thus, the question remains: does the resulting PC primarily shield the NP's inherent fibrillation-modulating properties, or can it be engineered to enhance targeting? Crucially, the PC can also act as a scaffold, where surface-induced crowding may unintentionally promote aggregation. Thus, beyond simple inhibition, NP probes can be utilized as a strategic platform to manage this nano-bio self-assembly. Such a perspective enables a transition from treating the PC as a biological barrier to utilizing it as an active, programmable tool for the advancement of nanomedicine.

## 5 Conclusions and future perspectives

The development of nanoparticles is increasingly evolving in broad range of clinical applications, holding promises to overcome limitations of drugs or therapeutics and navigate systemic, microenvironmental and cellular biological barriers. Nanoparticle-based therapies have the potential to offer several benefits, including more precise drug delivery, improved solubility, prevention of drug degradation, enhanced therapeutic efficacy, and reduced immune response. All of this can be achieved by engineering nanosystems to finely tune their peculiar physico-chemical properties, such as shape, size, charge, hydrophobicity, and surface features. However, manufacturing and quality control, clinical or regulatory hurdles and biological or physiological barriers, are challenging the nanomedicine and nanoparticle-based therapeutics.

As discussed herein, the biomolecular corona, mostly consisting of proteins but also of other types of biomolecules, form spontaneously upon delivery of NPs in biological tissues or fluids, thereby ultimately determining the fate of nanomaterials. Therefore, the development of safe and efficient nanoparticle-based diagnostics and therapeutics necessitates robust nano-bio information and reliable characterization of the protein corona.

Indeed, in the protein corona research, lack of standard methodology poses a challenge in the reproducibility and transparency of nanomedicine reports. Despite a milestone success, there is still a need for robust and accurate methodological approaches that can be reproduced in the wider research community. Thus, the application of appropriate characterization techniques during the preparation of the protein corona, and tailoring analytical methods and testing to specific nanoparticle designs or formulations helps create better nanoparticle medicines. Furthermore, strategies that are appropriate to actively controlling and exploiting protein corona properties demand protein corona engineering and advancing *in situ* characterization techniques. These integrated approaches will help to accelerate the achievement of safer and more effective biomedical applications.

Finally, successful clinical translation of nanomedicine technologies (*i.e.* timely and effective success) necessitates the integrated functioning of all stakeholders including regulatory agencies, funding agencies and the scientific community.

## Author contributions

G. S. and G. M. conceived the review topic and outline. G. S., Z. M., M. D. V., G. C., and M. M. performed the literature search and data collection. G. S., M. D., Z. M., and A. G. T. drafted the initial manuscript. G. S. and M. D. prepared the figures. G.S., G. D., L. R., R. F., C. I., and G. M. provided critical revisions and technical editing. All authors have read and agreed to the published version of the manuscript.

## Conflicts of interest

The authors declare that there are no conflicts of interest.

## Abbreviations

AFM: Atomic Force Microscopy
BCA: Bicinchoninic Acid Assay
BSA: Bovine Serum Albumin
CIEF-MS: Capillary isoelectric focusing-mass spectrometry
DLS: Dynamic Light Scattering
DSC: Differential Scanning Calorimetry
EM: Electron Microscopy
FCCS: Fluorescence Cross-Correlation Spectroscopy
FCS: Fluorescence Correlation Spectroscopy
FTIR: Fourier-Transform Infrared Spectroscopy
LC-MS: Liquid Chromatography-Tandem Mass Spectrometry
NP: Nanoparticle
PC: Protein corona
TGA: Thermogravimetric Analysis
TEM: Transmission electron microscopy
UV-VIS: Ultraviolet-Visible Spectroscopy
XPS: X-ray Photoelectron Spectroscopy
X-Ray: X-ray Diffraction (XRD)
ZETA: Zeta Potential

## Data Availability

This review paper does not contain any unpublished data. All figures included were created with https://www.biorender.com/, with appropriate citations and permissions secured. No new data were generated or analyzed for this work.

## References

[cit1] Dai H., Fan Q., Wang C. (2022). Exploration.

[cit2] Liu R., Luo C., Pang Z., Zhang J., Ruan S., Wu M., Wang L., Sun T., Li N., Han L. (2023). Chin. Chem. Lett..

[cit3] Zelmer C., Zweifel L. P., Kapinos L. E., Craciun I., Güven Z. P., Palivan C. G., Lim R. Y. (2020). Proc. Natl. Acad. Sci. U. S. A..

[cit4] Riccardi C., Napolitano F., Montesarchio D., Sampaolo S., Melone M. A. B. (2021). Pharmaceutics.

[cit5] Kumar A., Shahvej S., Yadav P., Modi U., Yadav A. K., Solanki R., Bhatia D. (2025). Pharmaceutics.

[cit6] Mitchell M. J., Billingsley M. M., Haley R. M., Wechsler M. E., Peppas N. A., Langer R. (2021). Nat. Rev. Drug Discovery.

[cit7] Chou W.-C., Lin Z. (2024). Curr. Opin. Biotechnol..

[cit8] Younis M. A., Sato Y., Kimura S., Harashima H. (2025). RSC Pharm..

[cit9] Berger S., Berger M., Bantz C., Maskos M., Wagner E. (2022). Biophys. Rev..

[cit10] Vilanova O., Martinez-Serra A., Monopoli M. P., Franzese G. (2025). Front. Nanotechnol..

[cit11] Panico S., Capolla S., Bozzer S., Toffoli G., Dal Bo M., Macor P. (2022). Pharmaceutics.

[cit12] Canchola A., Li K., Chen K., Borboa-Pimentel A., Chou C., Dela Rama R., Chen C.-Y., Chen X., Strobel M., Riviere J. E. (2025). ACS Nano.

[cit13] Mohammad-Beigi H., Zanganeh M. (2022). ACS nano.

[cit14] Chen P., Ding F., Cai R., Javed I., Yang W., Zhang Z., Li Y., Davis T. P., Ke P. C., Chen C. (2020). Nano today.

[cit15] Sharma A., Ghosh K. S. (2023). Bionanoscience.

[cit16] Bing J., Xiao X., McClements D. J., Biao Y., Chongjiang C. (2021). Food Hydrocolloids.

[cit17] Barbalinardo M., Caicci F., Cavallini M., Gentili D. (2018). Compr. Rev. Food Sci. Food Saf..

[cit18] Overby C., Park S., Summers A., Benoit D. S. W. (2023). Bioact. Mater..

[cit19] Mishra R. K., Ahmad A., Vyawahare A., Alam P., Khan T. H., Khan R. (2021). Int. J. Biol. Macromol..

[cit20] Liang L., Everest-Dass A. V., Kostyuk A. B., Khabir Z., Zhang R., Trushina D. B., Zvyagin A. V. (2022). Cells.

[cit21] Baimanov D., Wang L., Liu K., Pan M., Cai R., Yuan H., Huang W., Yuan Q., Zhou Y., Chen C., Zhao Y. (2023). Nanoscale Horiz..

[cit22] Nierenberg D., Khaled A. R., Flores O. (2018). Rep. Pract. Oncol. Radiother..

[cit23] Ashkarran A. A., Gharibi H., Grunberger J. W., Saei A. A., Khurana N., Mohammadpour R., Ghandehari H., Mahmoudi M. (2023). ACS Bio Med Chem Au.

[cit24] Prawatborisut M., Oberländer J., Jiang S., Graf R., Avlasevich Y., Morsbach S., Crespy D., Mailänder V. (2022). Small.

[cit25] Tenzer S. , Docter D., Kuharev J., Musyanovych A., Fetz V., Hecht R., Schlenk F., Fischer D., Kiouptsi K. and Reinhardt C., in Nano-enabled Medical Applications, Jenny Stanford Publishing, 2020, pp. 251–278

[cit26] Conjeevaram S. B., Blanchard R. M., Kadaba A., Adjei I. M. (2022). Nanoscale Adv..

[cit27] Nandakumar A., Wei W., Siddiqui G., Tang H., Li Y., Kakinen A., Wan X., Koppel K., Lin S., Davis T. P. (2021). ACS Appl. Mater. Interfaces.

[cit28] Salem S. S., Hammad E. N., Mohamed A. A., El-Dougdoug W. (2022). Biointerface Res. Appl. Chem..

[cit29] Hajipour M. J., Safavi-Sohi R., Sharifi S., Mahmoud N., Ashkarran A. A., Voke E., Serpooshan V., Ramezankhani M., Milani A. S., Landry M. P., Mahmoudi M. (2023). Small.

[cit30] Eker F., Duman H., Akdaşçi E., Bolat E., Sarıtaş S., Karav S., Witkowska A. M. (2024). Molecules.

[cit31] Cooley M., Sarode A., Hoore M., Fedosov D. A., Mitragotri S., Gupta A. S. (2018). Nanoscale.

[cit32] Khor S. Y., Vu M. N., Pilkington E. H., Johnston A. P., Whittaker M. R., Quinn J. F., Truong N. P., Davis T. P. (2018). Small.

[cit33] Dolai J., Mandal K., Jana N. R. (2021). ACS Appl. Nano Mater..

[cit34] Piella J., Bastús N. G., Puntes V. (2017). Bioconjugate Chem..

[cit35] Li F., Wang Y., Chen D., Du Y. (2024). Int. J. Mol. Sci..

[cit36] Panczyk T., Wolski P., Nieszporek K. (2025). J. Phys. Chem. B.

[cit37] Liu S., Wang Z., Jiang X., Gan J., Tian X., Xing Z., Yan Y., Chen J., Zhang J., Wang C. (2021). Biomaterials.

[cit38] Gerasimovich E., Karaulov A., Nabiev I., Sukhanova A. (2025). Nanomaterials.

[cit39] Yin Y.-W., Ma Y.-Q., Ding H.-M. (2024). Langmuir.

[cit40] Wang W., Huang Z., Li Y., Wang W., Shi J., Fu F., Huang Y., Pan X., Wu C. (2021). Acta Pharm. Sin. B.

[cit41] Zhao Z., Li G., Liu Q. S., Liu W., Qu G., Hu L., Long Y., Cai Z., Zhao X., Jiang G. (2021). J. Hazard. Mater..

[cit42] Pan D., Feng W., Li R., Lu Y., Qin M., Yang X., Xu Z. (2026). Langmuir.

[cit43] Wang W., Huang Z., Li Y., Wang W., Shi J., Fu F., Huang Y., Pan X., Wu C. (2021). Acta Pharm. Sin. B.

[cit44] Cedervall T., Lynch I., Lindman S., Berggård T., Thulin E., Nilsson H., Dawson K. A., Linse S. (2007). Proc. Natl. Acad. Sci. U. S. A..

[cit45] Yu J., Kim H.-J., Go M.-R., Bae S.-H., Choi S.-J. (2017). Nanomaterials.

[cit46] Marichal L., Klein G., Armengaud J., Boulard Y., Chédin S., Labarre J., Pin S., Renault J.-P., Aude J.-C. (2020). Nanomaterials.

[cit47] Partikel K., Korte R., Stein N. C., Mulac D., Herrmann F. C., Humpf H.-U., Langer K. (2019). Eur. J. Pharm. Biopharm..

[cit48] Lima T., Bernfur K., Vilanova M., Cedervall T. (2020). Sci. Rep..

[cit49] Wilhelm S., Tavares A. J., Dai Q., Ohta S., Audet J., Dvorak H. F., Chan W. C. (2016). Nat. Rev. Mater..

[cit50] von Roemeling C., Jiang W., Chan C. K., Weissman I. L., Kim B. Y. (2017). Trends Biotechnol..

[cit51] Gimondi S., de Castro J. V., Reis R. L., Ferreira H., Neves N. M. (2023). Colloids Surf., B.

[cit52] Nourani L., Lotfi A., Vand-Rajabpour H., Pourhashem Z., Nemati F., Mehrizi A. A. (2024). Mol. Biotechnol..

[cit53] Yu W., Liu R., Zhou Y., Gao H. (2020). ACS Cent. Sci..

[cit54] Beach M. A., Nayanathara U., Gao Y., Zhang C., Xiong Y., Wang Y., Such G. K. (2024). Chem. Rev..

[cit55] Parkatzidis K., Truong N. P., Rolland M., Lutz-Bueno V., Pilkington E. H., Mezzenga R., Anastasaki A. (2022). Angew. Chem., Int. Ed..

[cit56] Pinals R. L., Chio L., Ledesma F., Landry M. P. (2020). Analyst.

[cit57] Bilardo R., Traldi F., Vdovchenko A., Resmini M. (2022). Wiley Interdiscip. Rev.:Nanomed. Nanobiotechnol..

[cit58] Wang G., Wang W., Shangguan E., Gao S., Liu Y. (2020). Mater. Sci. Eng. C.

[cit59] Madathiparambil Visalakshan R., González García L. E., Benzigar M. R., Ghazaryan A., Simon J., Mierczynska-Vasilev A., Michl T. D., Vinu A., Mailänder V., Morsbach S. (2020). Small.

[cit60] Mosquera J. s., García I., Henriksen-Lacey M., Martínez-Calvo M., Dhanjani M. n., Mascareñas J. L., Liz-Marzán L. M. (2020). ACS Nano.

[cit61] Olfati A.-H., Safary A., Akbarzadeh-Khiavi M., Adibkia K. (2023). J. Drug Delivery Sci. Technol..

[cit62] Lu X., Xu P., Ding H.-M., Yu Y.-S., Huo D., Ma Y.-Q. (2019). Nat. Commun..

[cit63] Zheng L.-T., Yan Z.-S., Li X.-Y., Chang J.-J., Tan X.-Q., Wang Y.-X., Ding H.-M., Liu Q., Ma Y.-Q., Huo D. (2025). Nat. Commun..

[cit64] Kokkinopoulou M., Simon J., Landfester K., Mailänder V., Lieberwirth I. (2017). Nanoscale.

[cit65] Feiner-Gracia N., Beck M., Pujals S., Tosi S., Mandal T., Buske C., Linden M., Albertazzi L. (2017). Small.

[cit66] Kihara S., Ghosh S., McDougall D. R., Whitten A. E., Mata J. P., Köper I., McGillivray D. J. (2020). Biointerphases.

[cit67] Bashiri G., Padilla M. S., Swingle K. L., Shepherd S. J., Mitchell M. J., Wang K. (2023). Lab Chip.

[cit68] Lee H. (2020). Small.

[cit69] Oberländer J., Champanhac C., da Costa Marques R., Landfester K., Mailänder V. (2022). Acta Biomater..

[cit70] Martínez-Negro M., González-Rubio G., Aicart E., Landfester K., Guerrero-Martínez A., Junquera E. (2021). Adv. Colloid Interface Sci..

[cit71] Rabel M., Warncke P., Thürmer M., Grüttner C., Bergemann C., Kurland H. D., Müller F. A., Koeberle A., Fischer D. (2021). Nanoscale.

[cit72] Spreen H., Behrens M., Mulac D., Humpf H. U., Langer K. (2021). Eur. J. Pharm. Biopharm..

[cit73] Bewersdorff T., Gruber A., Eravci M., Dumbani M., Klinger D., Haase A. (2019). Int. J. Nanomed..

[cit74] Mekseriwattana W., Thiangtrongjit T., Reamtong O., Wongtrakoongate P., Katewongsa K. P. (2022). ACS Omega.

[cit75] Bertrand N., Grenier P., Mahmoudi M., Lima E. M., Appel E. A., Dormont F., Lim J.-M., Karnik R., Langer R., Farokhzad O. C. (2017). Nat. Commun..

[cit76] Nienhaus K., Nienhaus G. U. (2023). Small.

[cit77] Pakki S. P., Goyal S., Chinmay R., Chauhan V., Bendi A. (2025). J. Mater. Res..

[cit78] Pizzi D., Nandakumar A., Morrow J. P., Humphries J., Siddiqui G., Creek D. J., Quinn J. F., Yin J., Shi Q., Cheng W. (2024). Eur. Polym. J..

[cit79] Wang Y., Zhang X., Xie D., Chen C., Huang Z., Li Z. A. (2025). Adv. Funct. Mater..

[cit80] Kehr N. S., Jose J. (2017). Appl. Surf. Sci..

[cit81] Xue M.-D., Yin Y.-w., Zheng L., Li X., Tang W., Jin X., Zhang X., Zhao H., Ma Y.-q., Ding H.-M. (2025). Nano Lett..

[cit82] Liu F., Li X., Sheng A., Shang J., Wang Z., Liu J. (2019). Environ. Sci. Technol..

[cit83] Yu Y.-S., Tan R.-R., Zhu D., Ding H.-M. (2026). Langmuir.

[cit84] Hajipour M. J., Aghaverdi H., Serpooshan V., Vali H., Sheibani S., Mahmoudi M. (2021). Nat. Commun..

[cit85] Serpooshan V., Sheibani S., Pushparaj P., Wojcik M. (2018). ACS Nano.

[cit86] Hajipour M. J., Safavi-Sohi R., Sharifi S., Mahmoud N., Ashkarran A. A., Voke E., Serpooshan V., Ramezankhani M., Milani A. S., Landry M. P. (2023). Small.

[cit87] Prawatborisut M., Jiang S., Oberländer J., Mailänder V., Crespy D., Landfester K. (2022). Adv. Funct. Mater..

[cit88] Gomari M. M., Tarighi P., Choupani E., Abkhiz S., Mohamadzadeh M., Rostami N., Sadroddiny E., Baammi S., Uversky V. N., Dokholyan N. V. (2023). Int. J. Biol. Macromol..

[cit89] Ghosh G., Panicker L. (2021). Soft Matter.

[cit90] Mahmoudi M., Abdelmonem A. M., Behzadi S., Clement J. H., Dutz S., Ejtehadi M. R., Hartmann R., Kantner K., Linne U., Maffre P. (2013). ACS Nano.

[cit91] Satzer P., Svec F., Sekot G., Jungbauer A. (2016). Eng. Life Sci..

[cit92] Park S. J. (2020). Int. J. Nanomed..

[cit93] Mamdouh Z., Giocondi M.-C., Laprade R., Le Grimellec C. (1996). Biochim. Biophys. Acta, Biomembr..

[cit94] Hong G., Wu J. Z., Robinson J. T., Wang H., Zhang B., Dai H. (2012). Nat. Commun..

[cit95] Ge Y., Fu F., Gao Y., He T., Mailänder V., Crespy D., Landfester K., Jiang S. (2025). Advanced Science.

[cit96] Gorshkov V., Bubis J. A., Solovyeva E. M., Gorshkov M. V., Kjeldsen F. (2019). Environ. Sci.: Nano.

[cit97] Huang R., Carney R. P., Stellacci F., Lau B. L. (2013). Nanoscale.

[cit98] Rajendran D., Chandrasekaran N., Waychal Y., Mukherjee A. (2022). NanoImpact.

[cit99] Meesaragandla B., García I., Biedenweg D., Toro-Mendoza J., Coluzza I., Liz-Marzán L. M., Delcea M. (2020). Phys. Chem. Chem. Phys..

[cit100] Dewald I., Isakin O., Schubert J., Kraus T., Chanana M. (2015). J. Phys. Chem. C.

[cit101] Maity A., Bagchi D., Tabassum H., Nath P., Sinha S., Chakraborty A. (2024). J. Phys. Chem. B.

[cit102] Del Caño R., Mateus L., Sánchez-Obrero G., Sevilla J. M., Madueño R., Blázquez M., Pineda T. (2017). J. Colloid Interface Sci..

[cit103] Tingting H., Zhixiong L., Jiawei C. (2023). Rock Miner. Anal..

[cit104] Dar A. I., Randhawa S., Verma M., Saini T. C., Acharya A. (2025). Adv. Colloid Interface Sci..

[cit105] Mahmoudi M. (2022). Nat. Commun..

[cit106] Palchetti S., Pozzi D., Capriotti A. L., Barbera G., Chiozzi R. Z., Digiacomo L., Peruzzi G., Caracciolo G., Laganà A. (2017). Colloids Surf., B.

[cit107] Barbalinardo M., Bertacchini J., Bergamini L., Magarò M. S., Ortolani L., Sanson A., Palumbo C., Cavallini M., Gentili D. (2021). Nanoscale Adv..

[cit108] Conage-Pough J. E., Stopka S. A., Oh J. H., Mladek A. C., Burgenske D. M., Regan M. S., Baquer G., Decker P. A., Carlson B. L., Bakken K. K., Zhang J., Liu L., Sun C., Mu Z., Zhong W., Tran N. L., Elmquist W. F., Agar N. Y. R., Sarkaria J. N., White F. M. (2023). Neurooncol Adv..

[cit109] Nandakumar A., Xing Y., Aranha R. R., Faridi A., Kakinen A., Javed I., Koppel K., Pilkington E. H., Purcell A. W., Davis T. P. (2020). Biomacromolecules.

[cit110] Wang C., Chen B., He M., Hu B. (2021). ACS Nano.

[cit111] Mohammad-Beigi H., Zanganeh M., Scavenius C., Eskandari H., Farzadfard A., Shojaosadati S. A., Enghild J. J., Otzen D. E., Buell A. K., Sutherland D. S. (2022). ACS Nano.

[cit112] Li Q., Wen J., Yan Z., Sun H., Song E., Song Y. (2023). Langmuir.

[cit113] D'Onofrio M., Munari F., Assfalg M. (2020). Molecules.

[cit114] Li H., Wang N., Mao X. (2025). Nano Res..

[cit115] Da Silva-Candal A., Brown T., Krishnan V., Lopez-Loureiro I., Ávila-Gómez P., Pusuluri A., Pérez-Díaz A., Correa-Paz C., Hervella P., Castillo J. (2019). J. Controlled Release.

[cit116] Johnsen K. B., Bak M., Melander F., Thomsen M. S., Burkhart A., Kempen P. J., Andresen T. L., Moos T. (2019). J. Controlled Release.

[cit117] Zhang Y., Meng S., Ding J., Peng Q., Yu Y. (2019). Analyst.

[cit118] Szekeres G. P., Kneipp J. (2018). Analyst.

[cit119] Dobrovolskaia M. A., Neun B. W., Man S., Ye X., Hansen M., Patri A. K., Crist R. M., McNeil S. E. (2014). Nanomed. Nanotechnol. Biol. Med..

[cit120] Cox A., Andreozzi P., Dal Magro R., Fiordaliso F., Corbelli A., Talamini L., Chinello C., Raimondo F., Magni F., Tringali M. (2018). ACS Nano.

[cit121] Nicoletti M., Capodanno C., Gambarotti C., Fasoli E. (2018). Biochim. Biophys. Acta, Bioenerg..

[cit122] Yue Y., Behra R., Sigg L., Suter M. J.-F., Pillai S., Schirmer K. (2016). Environ. Sci.: Nano.

[cit123] Mekseriwattana W., Thiangtrongjit T., Reamtong O., Wongtrakoongate P., Katewongsa K. P. (2022). ACS Omega.

[cit124] Sebak A. A., Gomaa I. E. O., ElMeshad A. N., Farag M. H., Breitinger U., Breitinger H.-G., AbdelKader M. H. (2020). Int. J. Nanomed..

[cit125] Sheibani S., Basu K., Farnudi A., Ashkarran A., Ichikawa M., Presley J. F., Bui K. H., Ejtehadi M. R., Vali H., Mahmoudi M. (2021). Nat. Commun..

[cit126] Moghimi S. M., Simberg D. (2022). Nano Today.

[cit127] von Mentzer U., Selldén T., Råberg L., Erensoy G., Hultgård Ekwall A. K., Stubelius A. (2022). Osteoarthr. Cartil..

[cit128] Schöttler S., Klein K., Landfester K., Mailänder V. (2016). Nanoscale.

[cit129] Hoang K. N. L., Wheeler K. E., Murphy C. J. (2022). Anal. Chem..

[cit130] Docter D., Distler U., Storck W., Kuharev J., Wünsch D., Hahlbrock A., Knauer S. K., Tenzer S., Stauber R. H. (2014). Nat. Protoc..

[cit131] Winzen S., Schoettler S., Baier G., Rosenauer C., Mailaender V., Landfester K., Mohr K. (2015). Nanoscale.

[cit132] Brückner M., Simon J., Jiang S., Landfester K., Mailänder V. (2020). Acta Biomater..

[cit133] Hossen M. N., Elechalawar C. K., Sjoelund V., Moore K., Mannel R., Bhattacharya R., Mukherjee P. (2021). Cancer Nanotechnol..

[cit134] Durán N., Martinez D. S., Justo G. Z., de Lima R., de Castro V. L., Umbuzeiro G. A., Barbieri E., Durán M., Melo P. S., Alves O. L. (2015). J. Phys.: Conf. Ser..

[cit135] Franqui L. S., De Farias M. A., Portugal R. V., Costa C. A. R., Domingues R. R., Souza Filho A. G., Coluci V. R., Leme A. F. P., Martinez D. S. T. (2019). Mater. Sci. Eng. C..

[cit136] Partikel K., Korte R., Mulac D., Humpf H.-U., Langer K. (2019). Beilstein J. Nanotechnol..

[cit137] Li Y., Lee J.-S. (2020). Materials.

[cit138] Gan N., Sun Q., Zhao L., Tang P., Suo Z., Zhang S., Zhang Y., Zhang M., Wang W., Li H. (2019). Int. J. Biol. Macromol..

[cit139] Hoang K. N. L. (2022). ACS Omega.

[cit140] Bonvin D., Chiappe D., Moniatte M., Hofmann H., Mionić Ebersold M. (2017). Analyst.

[cit141] Qiu L., Zhang Y., Wei G., Wang C., Zhu Y., Yang T., Chu Z., Gao P., Cheng G., Ma A., Kwan Wong Y., Zhang J., Xu C., Wang J., Tang H. (2023). J. Colloid Interface Sci..

[cit142] Barbero F., Michelini S., Moriones O. H., Patarroyo J., Rosell J., M F. G., Vitali M., Martín L., Canals F., Duschl A., Horejs-Hoeck J., Mondragón L., Bastús N. G., Puntes V. (2022). Bioconjugate Chem..

[cit143] Hasan M., Gulzar H., Zafar A., ul Haq A., Mustafa G., Tariq T., Khalid A., Mahmmod A., Shu X., Mahmood N. (2021). Colloids Surf., B.

[cit144] Hasan M., Zafar A., Jabbar M., Tariq T., Manzoor Y., Ahmed M. M., Hassan S. G., Shu X., Mahmood N. (2022). Molecules.

[cit145] Pederzoli F., Tosi G., Genovese F., Belletti D., Vandelli M. A., Ballestrazzi A., Forni F., Ruozi B. (2018). Nanomedicine.

[cit146] Szekeres G. P., Fernández-Iglesias N., Kneipp J., Montes-Bayón M., Bettmer J. (2020). J. Proteomics Bioinf..

[cit147] Singh P., Szigyártó I. C., Ricci M., Zsila F., Juhász T., Mihály J., Bősze S., Bulyáki É., Kardos J., Kitka D., Varga Z., Beke-Somfai T. (2020). Front. Chem..

[cit148] Tóth E., Turiák L., Visnovitz T., Cserép C., Mázló A., Sódar B. W., Försönits A. I., Petővári G., Sebestyén A., Komlósi Z., Drahos L., Kittel Á., Nagy G., Bácsi A., Dénes Á., Gho Y. S., Szabó-Taylor K., Buzás E. I. (2021). J. Extracell. Vesicles.

[cit149] Qin M., Zhang J., Li M., Yang D., Liu D., Song S., Fu J., Zhang H., Dai W., Wang X., Wang Y., He B., Zhang Q. (2020). Theranostics.

[cit150] Ashkarran A. A., Dararatana N., Crespy D., Caracciolo G., Mahmoudi M. (2020). Nanoscale.

[cit151] Faserl K., Chetwynd A. J., Lynch I., Thorn J. A., Lindner H. H. (2019). Nanomaterials.

[cit152] Ghaffari B. , Grumelot S., Sadeghi S. A., Alpaydin A., Hilsen K., Shango B., Ritz D., Schmidt A., Vali H. and Sun L., bioRxiv: the Preprint Server for Biology, 2026, 2026.2002. 2019.706828

[cit153] Sakulkhu U., Mahmoudi M., Maurizi L., Salaklang J., Hofmann H. (2014). Sci. Rep..

[cit154] Zhang H., Peng J., Li X., Liu S., Hu Z., Xu G., Wu R. a. (2018). Colloids Surf., B.

[cit155] Weiss A. C. G., Krüger K., Besford Q. A., Schlenk M., Kempe K., Förster S., Caruso F. (2019). ACS Appl. Mater. Interfaces.

[cit156] Fu F., Crespy D., Landfester K., Jiang S. (2024). Chem. Soc. Rev..

[cit157] Fu F., Crespy D., Landfester K., Jiang S. (2024). Chem. Soc. Rev..

[cit158] Wu X., Tan F., Cheng S., Chang Y., Wang X., Chen L. (2022). Nanoscale.

[cit159] Pinals R. L., Yang D., Rosenberg D. J., Chaudhary T., Crothers A. R., Iavarone A. T., Hammel M., Landry M. P. (2020). Angew. Chem..

[cit160] Marichal L., Degrouard J., Gatin A., Raffray N., Aude J. C., Boulard Y., Combet S., Cousin F., Hourdez S., Mary J., Renault J. P., Pin S. (2020). Langmuir.

[cit161] Wang J., Zheng X., Zhang H. (2019). Spectrochim. Acta, Part A.

[cit162] Wang J., Yang B., Yu X., Chen S., Li W., Hong X. (2023). Chem.-Biol. Interact..

[cit163] Zhang T., Tang M., Yao Y., Ma Y., Pu Y. (2019). Proc. Natl. Acad. Sci. U. S. A..

[cit164] Preeyanka N., Akhuli A., Dey H., Chakraborty D., Rahaman A., Sarkar M. (2022). Langmuir.

[cit165] Sun N., Bai S., Dai L., Jia Y. (2024). Int. J. Mol. Sci..

[cit166] Carvalho J. T. T., Malfatti-Gasperini A., Boyd B. J., Wang L., Cardoso M. B. (2025). ACS Omega.

[cit167] Zhang Z., Ren J., Dai W., Zhang H., Wang X., He B., Zhang Q. (2023). Adv. Mater..

[cit168] Tabatabaeian Nimavard R., Sadeghi S. A., Mahmoudi M., Zhu G., Sun L. (2025). J. Am. Soc. Mass Spectrom..

[cit169] Zhu G., Sadeghi S. A., Mahmoudi M., Sun L. (2024). Chem. Commun..

[cit170] Blume J. E., Manning W. C., Troiano G., Hornburg D., Figa M., Hesterberg L., Platt T. L., Zhao X., Cuaresma R. A., Everley P. A. (2020). Nat. Commun..

[cit171] Gorohovs M., Dekhtyar Y. (2025). Molecules.

[cit172] Pederzoli F., Tosi G., Vandelli M. A., Belletti D., Forni F., Ruozi B. (2017). Wiley Interdiscip. Rev.:Nanomed. Nanobiotechnol..

[cit173] Fischer K., Schmidt M. (2016). Biomaterials.

[cit174] Kopac T. (2021). Int. J. Biol. Macromol..

[cit175] Jafari S., Izadi Z., Alaei L., Jaymand M., Samadian H., Kashani V. O., Derakhshankhah H., Hayati P., Noori F., Mansouri K., Moakedi F., Janczak J., Soltanian Fard M. J., Fayaz Bakhsh N. (2020). Sci. Rep..

[cit176] Yin M. M., Chen W. Q., Lu Y. Q., Han J. Y., Liu Y., Jiang F. L. (2020). Nanoscale.

[cit177] Ciobanu V., Roncari F., Ceccone G., Braniste T., Ponti J., Bogni A., Guerrini G., Cassano D., Colpo P., Tiginyanu I. (2022). J. Appl. Biomater. Funct. Mater..

[cit178] Krohn J.-H., Mamot A., Kaletta N., Qutbuddin Y., Schwille P. (2025). Biophys. J..

[cit179] Lima A. F., Guido V. S., Mina N., Torquato R. J. S., Sousa A. A. (2023). Langmuir.

[cit180] Sebak A. A., Gomaa I. E. O., ElMeshad A. N., Farag M. H., Breitinger U., Breitinger H. G., AbdelKader M. H. (2020). Int. J. Nanomed..

[cit181] Della Valle M., D'Abrosca G., Gentile M., Russo L., Isernia C., Di Gaetano S., Avolio R., Castaldo R., Cocca M., Gentile G. (2022). Chem. Sci..

[cit182] Lee H. (2024). Pharmaceutics.

[cit183] Tang H., Wang J., Mahmoudi M. (2025). Nat. Protoc..

[cit184] Toby T. K., Fornelli L., Kelleher N. L. (2016). Annu. Rev. Anal. Chem..

[cit185] Mahmoudi M., Sadeghi S., Li K., Yue Y., Nimavard R. T., Grumelot S., Saei A., Vali H., Liu X., Sun L. (2025). Res. Sq..

[cit186] Gimondi S., Ferreira H., Reis R. L., Neves N. M. (2023). ACS Nano.

[cit187] Ban Z., Yuan P., Yu F., Peng T., Zhou Q., Hu X. (2020). Proc. Natl. Acad. Sci. U. S. A..

[cit188] Duan Y., Coreas R., Liu Y., Bitounis D., Zhang Z., Parviz D., Strano M., Demokritou P., Zhong W. (2020). NanoImpact.

[cit189] Sengottiyan S., Mikolajczyk A., Jagiełło K., Swirog M., Puzyn T. (2023). ACS Nano.

[cit190] Saei A. A., Sun L., Mahmoudi M. (2025). Proteomics.

[cit191] Ashkarran A. A., Gharibi H., Modaresi S. M., Saei A. A., Mahmoudi M. (2024). Nano Lett..

[cit192] Haro-Martinez E., Muscolino E., Moral N., Duran J., Fornaguera C. (2026). Drug Delivery Transl. Res..

[cit193] Jiménez A., Estudillo E., Guzmán-Ruiz M. A., Herrera-Mundo N., Victoria-Acosta G., Cortés-Malagón E. M., López-Ornelas A. (2025). Pharmaceutics.

[cit194] Haid M., Muschet C., Wahl S., Römisch-Margl W., Prehn C., Möller G., Adamski J. (2018). J. Proteome Res..

[cit195] Zhu D., Yan H., Zhou Z., Tang J., Liu X., Hartmann R., Parak W. J., Feliu N., Shen Y. (2018). Biomater. Sci..

[cit196] Wen X., Ou L., Cutshaw G., Uthaman S., Ou Y. C., Zhu T., Szakas S., Carney B., Houghton J., Gundlach-Graham A., Rafat M., Yang K., Bardhan R. (2023). Small.

[cit197] Schöttler S., Becker G., Winzen S., Steinbach T., Mohr K., Landfester K., Mailänder V., Wurm F. R. (2016). Nat. Nanotechnol..

[cit198] Bianco A., Li D., Wang F., Di H., Liu X., Zhang P., Zhou W., Liu D. (2019). ACS Appl. Mater. Interfaces.

[cit199] Pattipeiluhu R., Crielaard S., Klein-Schiphorst I., Florea B. I., Kros A., Campbell F. (2020). ACS Cent. Sci..

[cit200] Meewan J., Somani S., Laskar P. (2022). Pharmaceutics.

[cit201] Grundler J., Shin K., Suh H. W., Whang C.-H., Fulgoni G., Pierce R. W., Saltzman W. M. (2024). ACS Nano.

[cit202] Walkey C. D., Olsen J. B., Guo H., Emili A., Chan W. C. (2012). J. Am. Chem. Soc..

[cit203] Sieg H., Braeuning C., Kunz B. M., Daher H., Kästner C., Krause B. C., Meyer T., Jalili P., Hogeveen K., Böhmert L., Lichtenstein D., Burel A., Chevance S., Jungnickel H., Tentschert J., Laux P., Braeuning A., Gauffre F., Fessard V., Meijer J., Estrela-Lopis I., Thünemann A. F., Luch A., Lampen A. (2018). Nanotoxicology.

[cit204] Partikel K., Korte R., Stein N. C., Mulac D., Herrmann F. C., Humpf H. U., Langer K. (2019). Eur. J. Pharm. Biopharm..

[cit205] Kim H., Röth D., Isoe Y., Hayashi K., Mochizuki C., Kalkum M., Nakamura M. (2021). Nanoscale.

[cit206] Zhang P., Sun F., Liu S., Jiang S. (2016). J. Controlled Release.

[cit207] Mohamed M., Abu Lila A. S., Shimizu T., Alaaeldin E., Hussein A., Sarhan H. A., Szebeni J., Ishida T. (2019). Sci. Technol. Adv. Mater..

[cit208] Zhao J., Qin Z., Wu J., Li L., Jin Q., Ji J. (2018). Biomater. Sci..

[cit209] Sloan-Dennison S., Schultz Z. D. (2019). Chem. Sci..

[cit210] Tonigold M., Simon J., Estupiñán D., Kokkinopoulou M., Reinholz J., Kintzel U., Kaltbeitzel A., Renz P., Domogalla M. P., Steinbrink K. (2018). Nat. Nanotechnol..

[cit211] Zhang Z., Guan J., Jiang Z., Yang Y., Liu J., Hua W., Mao Y., Li C., Lu W., Qian J. (2019). Nat. Commun..

[cit212] Ma S., Gu C., Xu J., He J., Li S., Zheng H., Pang B., Wen Y., Fang Q., Liu W., Tian J. (2022). Int. J. Nanomed..

[cit213] Tekie F. S. M., Hajiramezanali M., Geramifar P., Raoufi M., Dinarvand R., Soleimani M., Atyabi F. (2020). Sci. Rep..

[cit214] Sebak A. A., Gomaa I. E. O., ElMeshad A. N., Farag M. H., Breitinger U., Breitinger H. G., AbdelKader M. H. (2020). Int. J. Nanomed..

[cit215] Giulimondi F., Vulpis E., Digiacomo L., Giuli M. V., Mancusi A., Capriotti A. L., Laganà A., Cerrato A., Zenezini Chiozzi R., Nicoletti C. (2022). ACS Nano.

[cit216] Li S., Ju Y., Zhou J., Faria M., Ang C.-S., Mitchell A. J., Zhong Q.-Z., Zheng T., Kent S. J., Caruso F. (2022). J. Mater. Chem. B.

[cit217] Bondžić A. M., Jovanović D., Arsenijević N., Laban B., Lazarević Pašti T., Klekotka U., Bondžić B. P. (2022). Int. J. Mol. Sci..

[cit218] Niaz T., Sarkar A., Mackie A., Imran M. (2021). Int. J. Biol. Macromol..

[cit219] Schöttler S., Becker G., Winzen S., Steinbach T., Mohr K., Landfester K., Mailänder V., Wurm F. R. (2016). Nat. Nanotechnol..

[cit220] Hadjidemetriou M., Kostarelos K. (2017). Nat. Nanotechnol..

[cit221] Maity A., Bagchi D., De S. K., Chakraborty A. (2023). Langmuir.

[cit222] Sanità G., Armanetti P., Silvestri B., Carrese B., Calì G., Pota G., Pezzella A., d'Ischia M., Luciani G., Menichetti L. (2020). Front. Bioeng. Biotechnol..

[cit223] Shen Y., Wang M., Wang H., Zhou J., Chen J. (2022). ACS Appl. Mater. Interfaces.

[cit224] Yang S., Zhang Y., Lu S., Liu L., Yang L., Guo Y., Yu S., Yang H. (2020). Colloids Surf., B.

[cit225] Stordy B., Zhang Y., Sepahi Z., Khatami M. H., Kim P. M., Chan W. C. (2022). Chem. Mater..

[cit226] Ji Y. M., Zhang W., Zhang J. D., Li X. F., Yu F. D., Li C. Y., Liu G. J. (2022). Nanoscale.

[cit227] Wu H., Wang M. D., Liang L., Xing H., Zhang C. W., Shen F., Huang D. S., Yang T. (2021). Small.

[cit228] Liu X., Li C., Lv J., Huang F., An Y., Shi L., Ma R. (2020). ACS Appl. Bio Mater..

[cit229] Poon W., Kingston B. R., Ouyang B., Ngo W., Chan W. C. (2020). Nat. Nanotechnol..

[cit230] Sindhwani S., Syed A. M., Ngai J., Kingston B. R., Maiorino L., Rothschild J., MacMillan P., Zhang Y., Rajesh N. U., Hoang T. (2020). Nat. Mater..

[cit231] Degors I. M., Wang C., Rehman Z. U., Zuhorn I. S. (2019). Acc. Chem. Res..

[cit232] Subramaniam S., Joyce P., Donnellan L., Young C., Wignall A., Hoffmann P., Prestidge C. A. (2023). J. Colloid Interface Sci..

[cit233] Ren J., Andrikopoulos N., Velonia K., Tang H., Cai R., Ding F., Ke P. C., Chen C. (2022). J. Am. Chem. Soc..

[cit234] Ritz S., Schöttler S., Kotman N., Baier G., Musyanovych A., Kuharev J. r., Landfester K., Schild H. r., Jahn O., Tenzer S. (2015). Biomacromolecules.

[cit235] Behzadi S., Serpooshan V., Tao W., Hamaly M. A., Alkawareek M. Y., Dreaden E. C., Brown D., Alkilany A. M., Farokhzad O. C., Mahmoudi M. (2017). Chem. Soc. Rev..

[cit236] Gehr P. and Zellner R., Biological Responses to Nanoscale Particles, Springer, 2019 10.3762/bjnano.6.37 PMC4362476 25821677

[cit237] Means N., Elechalawar C. K., Chen W. R., Bhattacharya R., Mukherjee P. (2022). Mol. Aspects Med..

[cit238] Kaksonen M., Roux A. (2018). Nat. Rev. Mol. Cell Biol..

[cit239] Bitsikas V., Corrêa Jr I. R., Nichols B. J. (2014). eLife.

[cit240] Hocking K. M., Evans B. C., Komalavilas P., Cheung-Flynn J., Duvall C. L., Brophy C. M. (2019). Tissue Eng., Part A.

[cit241] Yue J., Feliciano T. J., Li W., Lee A., Odom T. W. (2017). Bioconjugate Chem..

[cit242] Ho Y. T., Kamm R. D., Kah J. C. Y. (2018). Adv. Healthcare Mater..

[cit243] Johnson L. T., Zhang D., Zhou K., Lee S. M. (2022). Mol. Pharmaceutics.

[cit244] Conjeevaram S. B., Blanchard R. M. (2022). Nanoscale Adv..

[cit245] Nguyen V. H., Meghani N. M., Amin H. H., Tran T. T. D., Tran P. H. L., Park C., Lee B. J. (2018). ACS Nano.

[cit246] Ding L., Yao C., Yin X., Li C., Huang Y., Wu M., Wang B., Guo X., Wang Y., Wu M. (2018). Small.

[cit247] Li Y., Monteiro-Riviere N. A. (2016). Nanomedicine.

[cit248] Elechalawar C. K., Rao G., Gulla S. K., Patel M. M., Frickenstein A., Means N., Roy R. V., Tsiokas L., Asfa S., Panja P. (2023). ACS Nano.

[cit249] Carnovale C., Bryant G., Shukla R., Bansal V. (2019). ACS Omega.

[cit250] Foroozandeh P., Aziz A. A. (2018). Nanoscale Res. Lett..

[cit251] Leong J., Teo J. Y., Aakalu V. K., Yang Y. Y., Kong H. (2018). Adv. Healthcare Mater..

[cit252] Hou Y., Tu S., Zhao X., Li G., Li N., Zou A. (2023). Biochim. Biophys. Acta, Gen. Subj..

[cit253] Villaverde G., Baeza A. (2019). Beilstein J. Nanotechnol..

[cit254] Ahmad A., Khan F., Mishra R. K., Khan R. (2019). J. Med. Chem..

[cit255] Chen R., Huang Y., Wang L., Zhou J., Tan Y., Peng C., Yang P., Peng W., Li J., Gu Q. (2021). Biomater. Sci..

[cit256] Nakamura N., Ohta S. (2024). Curr. Opin. Biotechnol..

[cit257] Francia V., Montizaan D., Salvati A. (2020). Beilstein J. Nanotechnol..

[cit258] Mosquera J., García I., Henriksen-Lacey M., Martínez Calvo M., Dhanjani M., Mascareñas J. L., Liz-Marzán L. M. (2020). ACS Nano.

[cit259] Agnihotri T. G., Alexander A., Agrawal M., Dubey S. K., Jain A. (2023). ACS Appl. Bio Mater..

[cit260] Jeon S., Clavadetscher J., Lee D.-K., Chankeshwara S. V., Bradley M., Cho W.-S. (2018). Nanomaterials.

[cit261] Li W., Cao Z., Liu R., Liu L., Li H., Li X., Chen Y., Lu C., Liu Y. (2019). Artif. Cells, Nanomed., Biotechnol..

[cit262] Cai R., Ren J., Guo M., Wei T., Liu Y., Xie C., Zhang P., Guo Z., Chetwynd A. J., Ke P. C., Lynch I., Chen C. (2022). Proc. Natl. Acad. Sci. U. S. A..

[cit263] Wang Y., Zhang H., Xiao W., Liu Y., Zhou Y., He X., Xia X., Gong T., Wang L., Gao H. (2021). Nanotoxicology.

[cit264] Tran T. T., Roffler S. R. (2023). Curr. Opin. Biotechnol..

[cit265] Chen F., Wang G., Griffin J. I., Brenneman B., Banda N. K., Holers V. M., Backos D. S., Wu L., Moghimi S. M., Simberg D. (2017). Nat. Nanotechnol..

[cit266] Digiacomo L., Pozzi D., Palchetti S., Zingoni A., Caracciolo G. (2020). Wiley Interdiscip. Rev.:Nanomed. Nanobiotechnol..

[cit267] Willem M., Fändrich M. (2022). Science.

[cit268] Li D., Liu C. (2022). Nat. Rev. Neurosci..

[cit269] Geng H., Pan Y. c., Zhang R., Gao D., Wang Z., Li B., Li N., Guo D. s., Xing C. (2021). Adv. Funct. Mater..

[cit270] Prajapat M., Shekhar N., Sarma P., Avti P., Singh S., Kaur H., Bhattacharyya A., Kumar S., Sharma S., Prakash A., Medhi B. (2020). J. Mol. Graphics Modell..

[cit271] Chaki S., Santra S., Dasgupta S. (2024). J. Phys. Chem. B.

[cit272] Xu J., Ma X. P., Bai L., Wang M., Deng W., Ning N. (2020). Medicine.

[cit273] Fan W., Chen X.-d., Liu L.-m., Chen N., Zhou X.-g., Zhang Z.-h., Liu S.-l. (2021). Chin. J. Chem. Phys..

[cit274] Mitra A., Chakraborty D., Naik L., Dhiman R., Sarkar N. (2025). Int. J. Biol. Macromol..

[cit275] Ly P.-D., Ly K.-N., Phan H.-L., Nguyen H. H., Duong V.-A., Nguyen H. V. (2024). Front. Nanotechnol..

[cit276] Zeng H., Qi Y., Zhang Z., Liu C., Peng W., Zhang Y. (2021). Chin. Chem. Lett..

[cit277] Meisl G., Kirkegaard J. B., Arosio P., Michaels T. C., Vendruscolo M., Dobson C. M., Linse S., Knowles T. P. (2016). Nat. Protoc..

[cit278] Flint Z., Grannemann H., Baffour K., Koti N., Taylor E., Grier E., Sutton C., Johnson D., Dandawate P., Patel R. (2024). ACS Chem. Neurosci..

[cit279] Ke P. C., Pilkington E. H., Sun Y., Javed I., Kakinen A., Peng G., Ding F., Davis T. P. (2020). Adv. Mater..

[cit280] Yagi-Utsumi M., Kanaoka Y., Miyajima S., Itoh S. G., Yanagisawa K., Okumura H., Uchihashi T., Kato K. (2024). J. Am. Chem. Soc..

[cit281] Asthana S., Bhattacharyya D., Kumari S., Nayak P. S., Saleem M., Bhunia A., Jha S. (2020). Int. J. Biol. Macromol..

[cit282] Tsoi P. S., Lucas L., Rhoades D., Ferreon J. C., Ferreon A. C. M. (2025). Biomolecules.

[cit283] Wei J., Meisl G., Dear A. J., Michaels T. C., Knowles T. P. (2025). Annu. Rev. Biophys..

[cit284] Li L., Liu J., Li X., Tang Y., Shi C., Zhang X., Cui Y., Wang L., Xu W. (2022). Soft Matter.

[cit285] John T., Adler J., Elsner C., Petzold J., Krueger M., Martin L. L., Huster D., Risselada H. J., Abel B. (2022). J. Colloid Interface Sci..

[cit286] Ban D. K., Paul S. (2019). Appl. Surf. Sci..

[cit287] Sukhanova A., Poly S., Bozrova S., Lambert É., Ewald M., Karaulov A., Molinari M., Nabiev I. (2019). Front. Chem..

[cit288] Grigolato F., Arosio P. (2021). Biophys. Chem..

[cit289] Moore K. A., Pate K. M., Soto-Ortega D. D., Lohse S., van der Munnik N., Lim M., Jackson K. S., Lyles V. D., Jones L., Glassgow N. (2017). J. Biol. Eng..

[cit290] Sudhakar S., Mani E. (2019). Langmuir.

[cit291] Giannousi K., Geromichalos G., Kakolyri D., Mourdikoudis S., Dendrinou-Samara C. (2020). ACS Chem. Neurosci..

[cit292] Yang L., Sun J., Xie W., Liu Y., Liu J. (2017). J. Mater. Chem. B.

[cit293] Huang Y., Chang Y., Liu L., Wang J. (2021). Molecules.

[cit294] Zhao J., Xu N., Yang X., Ling G., Zhang P. (2022). Colloid Interface Sci. Commun..

[cit295] Feng Q., Wang N., Zhang X., Mei Y., Fu R., Chen J., Yuan X., Yang S., Zhang Z., Zhao H. (2023). Nano today.

[cit296] Sutherland D. S., Meesaragandla B., García I. (2020). Artif. Cells, Nanomed., Biotechnol..

[cit297] Zhang Y., Liu Y., Zhao W., Sun Y. (2021). Int. J. Biol. Macromol..

[cit298] Liu H., Xie B., Dong X., Zhang L., Wang Y., Liu F., Sun Y. (2016). React. Funct. Polym..

[cit299] Mirzaei-Behbahani B., Meratan A. A., Moosakhani B., Mohammad-Zaheri M., Mousavi-Jarrahi Z., Nikfarjam N., Shahsavani M. B., Saboury A. A. (2024). Sci. Rep..

[cit300] Fang S., Zhang K., Liu D., Yang Y., Xi H., Xie W., Diao K., Rao Z., Wang D., Yang W. (2024). Front. Nutr..

[cit301] Ban D. K., Paul S. (2016). ACS Appl. Mater. Interfaces.

[cit302] Kirichenko K. Y., Pamirsky I., Timkin P., Kotelnikov D., Pogodaev A., Chernousov V., Gridasov A., Kholodov A., Parshin S., Golokhvast K. (2025). BioNanoScience.

[cit303] Dubey K., Anand B. G., Badhwar R., Bagler G., Navya P. N., Daima H. K., Kar K. (2015). Amino Acids.

[cit304] Antosova A., Gancar M., Bednarikova Z., Marek J., Zahn D., Dutz S., Gazova Z. (2021). Biochim. Biophys. Acta, Gen. Subj..

[cit305] Sharma A., Kesamsetty D., Debnath J., Ghosh K. S. (2023). J. Mol. Liq..

[cit306] Heegaard P. M., Pedersen H. G., Flink J., Boas U. (2004). FEBS Lett..

[cit307] Bashirova N., Schölzel F., Hornig D., Scheidt H. A., Krueger M., Salvan G., Huster D., Matysik J., Alia A. (2025). Molecules.

[cit308] Bugge K., Staby L., Salladini E., Falbe-Hansen R. G., Kragelund B. B., Skriver K. (2021). J. Biol. Chem..

[cit309] Yang X., Zhang X., Yu G., Ma Y., Li X., Guo Y., Wang S., Ge D., Xue C., Jin K. (2025). ACS Appl. Mater. Interfaces.

[cit310] Slekiene N., Snitka V., Bruzaite I., Ramanavicius A. (2022). Materials.

[cit311] Wu R., Ou X., Zhang L., Wang F., Liu L. (2022). ChemBioChem.

[cit312] Samal P., Satpathy S., Panigrahi L. L., Jha S., Arakha M. (2025). Nanoscale Horiz..

[cit313] Lotfabadi A., Hajipour M. J., Derakhshankhah H., Peirovi A., Saffar S., Shams E., Fatemi E., Barzegari E., Sarvari S., Moakedi F., Ferdousi M., Atyabi F., Saboury A. A., Dinarvand R. (2018). ACS Chem. Neurosci..

[cit314] Pilkington E. H., Gustafsson O. J. R., Xing Y., Hernandez-Fernaud J., Zampronio C., Kakinen A., Faridi A., Ding F., Wilson P., Ke P. C., Davis T. P. (2018). ACS Nano.

[cit315] Zanganeh S., Spitler R., Erfanzadeh M., Alkilany A. M., Mahmoudi M. (2016). Int. J. Biochem. Cell Biol..

[cit316] Chen Z., Chen X., Huang J., Wang J., Wang Z. (2022). Biomimetics.

[cit317] Li J., Guo M., Tian X., Wang X., Yang X., Wu P., Liu C., Xiao Z., Qu Y., Yin Y., Wang C., Zhang Y., Zhu Z., Liu Z., Peng C., Zhu T., Liang Q. (2021). Med.

[cit318] Pilkington E. H., Xing Y., Wang B., Kakinen A., Wang M., Davis T. P., Ding F., Ke P. C. (2017). Sci. Rep..

[cit319] Mahmoudi M., Monopoli M. P., Rezaei M., Lynch I., Bertoli F., McManus J. J., Dawson K. A. (2013). ChemBioChem.

[cit320] Pilkington E. H., Lai M., Ge X., Stanley W. J., Wang B., Wang M., Kakinen A., Sani M.-A., Whittaker M. R., Gurzov E. N., Ding F., Quinn J. F., Davis T. P., Ke P. C. (2017). Biomacromolecules.

[cit321] Mirsadeghi S., Dinarvand R., Ghahremani M. H., Hormozi-Nezhad M. R., Mahmoudi Z., Hajipour M. J., Atyabi F., Ghavami M., Mahmoudi M. (2015). Nanoscale.

[cit322] Lotfabadi A., Hajipour M. J., Derakhshankhah H., Peirovi A., Saffar S., Shams E., Fatemi E., Barzegari E., Sarvari S., Moakedi F. (2018). ACS Chem. Neurosci..

[cit323] Ezzat K., Pernemalm M., Pålsson S., Roberts T. C., Järver P., Dondalska A., Bestas B., Sobkowiak M. J., Levänen B., Sköld M., Thompson E. A., Saher O., Kari O. K., Lajunen T., Sverremark Ekström E., Nilsson C., Ishchenko Y., Malm T., Wood M. J. A., Power U. F., Masich S., Lindén A., Sandberg J. K., Lehtiö J., Spetz A. L., El Andaloussi S. (2019). Nat. Commun..

