# Correction: The protein corona at the nano-bio interface: the need for standardized methodology and opportunities for neurodegenerative disease intervention

**DOI:** 10.1039/d6ra90063a

**Published:** 2026-07-06

**Authors:** Getasew Shitaye, Martina Dragone, Zewdie Mekonnen, Awet Ghebretinsae Tewelde, Maria della Valle, Gaetano Caputo, Mohammadhossein Mosalaeizadehyazd, Gianluca D'Abrosca, Luigi Russo, Roberto Fattorusso, Carla Isernia, Gaetano Malgieri

**Affiliations:** a Department of Environmental, Biological and Pharmaceutical Science and Technology, University of Campania “Luigi Vanvitelli” Caserta Italy getasewshitaye.ayalew@unicampania.it; b Department of Biochemistry, College of Medicine and Health Sciences, Bahir Dar University Bahir Dar Ethiopia getasew.shitaye@bdu.edu.et; c Department of Human Sciences, Link Campus University Roma Italy; d Institute of Crystallography, CNR Via Vivaldi, 43, 81100 Caserta Italy

## Abstract

Correction for ‘The protein corona at the nano-bio interface: the need for standardized methodology and opportunities for neurodegenerative disease intervention’ by Getasew Shitaye *et al.*, *RSC Adv.*, 2026, **16**, 27424–27452, https://doi.org/10.1039/D6RA01255H.

The authors regret the incorrect citation details for two references, ref. 17 and 107. Please find the correct references listed below as ref. 1 and 2, respectively.

The Royal Society of Chemistry apologises for these errors and any consequent inconvenience to authors and readers.
